# Loss of SNORA73 reprograms cellular metabolism and protects against steatohepatitis

**DOI:** 10.1038/s41467-021-25457-y

**Published:** 2021-09-01

**Authors:** Arthur C. Sletten, Jessica W. Davidson, Busra Yagabasan, Samantha Moores, Michaela Schwaiger-Haber, Hideji Fujiwara, Sarah Gale, Xuntian Jiang, Rohini Sidhu, Susan J. Gelman, Shuang Zhao, Gary J. Patti, Daniel S. Ory, Jean E. Schaffer

**Affiliations:** 1 Department of Medicine, Washington University in St. Louis, St. Louis, MO USA; 2 Joslin Diabetes Center, Harvard Medical School, Boston, MA USA; 3 Department of Chemistry, Washington University in St. Louis, St. Louis, MO USA

**Keywords:** Lipids, Small RNAs, Metabolic disorders

## Abstract

Dyslipidemia and resulting lipotoxicity are pathologic signatures of metabolic syndrome and type 2 diabetes. Excess lipid causes cell dysfunction and induces cell death through pleiotropic mechanisms that link to oxidative stress. However, pathways that regulate the response to metabolic stress are not well understood. Herein, we show that disruption of the box H/ACA SNORA73 small nucleolar RNAs encoded within the small nucleolar RNA hosting gene 3 *(Snhg3*) causes resistance to lipid-induced cell death and general oxidative stress in cultured cells. This protection from metabolic stress is associated with broad reprogramming of oxidative metabolism that is dependent on the mammalian target of rapamycin signaling axis. Furthermore, we show that knockdown of SNORA73 in vivo protects against hepatic steatosis and lipid-induced oxidative stress and inflammation. Our findings demonstrate a role for SNORA73 in the regulation of metabolism and lipotoxicity.

## Abstract (web summary)

Lipid induced stress contributes to metabolic diseases. Here the authors identify small nucleolar RNA 73 (SNORA73) in a screen for genes that protect against lipotoxicity and show that deficiency of SNORA73 reprograms oxidative metabolism and protects against steatohepatitis in mice.

## Introduction

In states of nutrient excess, lipid overload exceeds the capacity of adipose tissue to store triglycerides and the ability of non-adipose tissues to metabolize fatty acids. This leads to ectopic lipid storage in tissues such as the liver, heart, and skeletal muscle. Although triglyceride stores in non-adipose cells initially serve a cytoprotective role^1^, ectopic steatosis is ultimately associated with cell dysfunction and cell death that impairs organ function through the process of lipotoxicity^2^. Studies in animal models provide compelling evidence that lipotoxicity contributes to the pathogenesis of non-alcoholic fatty liver disease (NAFLD), the most common complication of type 2 diabetes and metabolic syndrome^3,4^.

Lipotoxicity is characterized by activation of stress response pathways as a consequence of excessive supply of substrates to physiological pathways of lipid utilization. Saturated fatty acids fuel the de novo synthetic pathway for ceramides, which can initiate signaling that leads to cell death^5^. Excessive lipid uptake, particularly saturated fatty acids, causes rapid remodeling of endoplasmic reticulum (ER) membranes that impairs organelle integrity and leads to activation of the ER stress response^6,7^. Mitochondrial dysfunction, precipitated by both adverse membrane remodeling and by augmented substrate metabolism, impairs energy production and initiates mitochondrial programs of apoptosis^8,9^. Both ER stress and mitochondrial dysfunction lead to production of reactive oxygen species (ROS) that overwhelm endogenous antioxidant mechanisms^10^. ROS propagation is further compounded by saturated fatty acid-induced activation of NADPH oxidase, NF-κB-mediated transcription of pro-inflammatory cytokines, and death receptor signaling^11–13^. The observation that antioxidants mitigate lipotoxicity supports the notion that oxidative stress is a critical factor that promotes lipotoxic cell death^14^. Systemically, the intersection of ER and oxidative stress signaling pathways with inflammatory signaling also leads to chronic low-grade inflammation^15^. Nonetheless, the proximal molecular transducers that control the lipotoxic response remain incompletely characterized.

Unbiased genetic screens have elucidated a number of regulators of the response to metabolic overload^16–19^. These studies have established important roles for enzymes in phospholipid and triglyceride synthesis pathways and protein modulators of lipid droplet function. Furthermore, through a retroviral promoter trap mutagenesis screen for palmitate-resistance in Chinese hamster ovary (CHO) cells, our lab has discovered noncoding RNAs that control responses to lipotoxic stress. These RNAs include the long noncoding RNA (lncRNA), Gadd7, and the box C/D snoRNAs encoded within the ribosomal protein L13a locus^20–22^. Herein, we describe a mutant cell line from this screen in which the locus encoding small nucleolar RNA hosting gene 3 (*Snhg3*) has been disrupted. This locus produces the lncRNA, SNHG3, and two highly conserved snoRNAs, SNORA73A and SNORA73B (U17A and U17B in prior nomenclature). While the molecular function of the lncRNA is not well understood, SNORA73A and SNORA73B direct one of several steps in the processing of pre-rRNAs to produce mature 18S, 5.8S, and 28S rRNAs^23^. We show that these snoRNAs, but not the SNHG3 lncRNA, are critical for metabolic stress responses and regulate cell metabolism through the mammalian target of rapamycin (mTOR) pathway. Our findings in cultured cells and in vivo elucidate a role for SNORA73 in the regulation of metabolic stress.

## Results

### *Snhg3* mutants are resistant to lipotoxic cell death

**Fig. 1:**
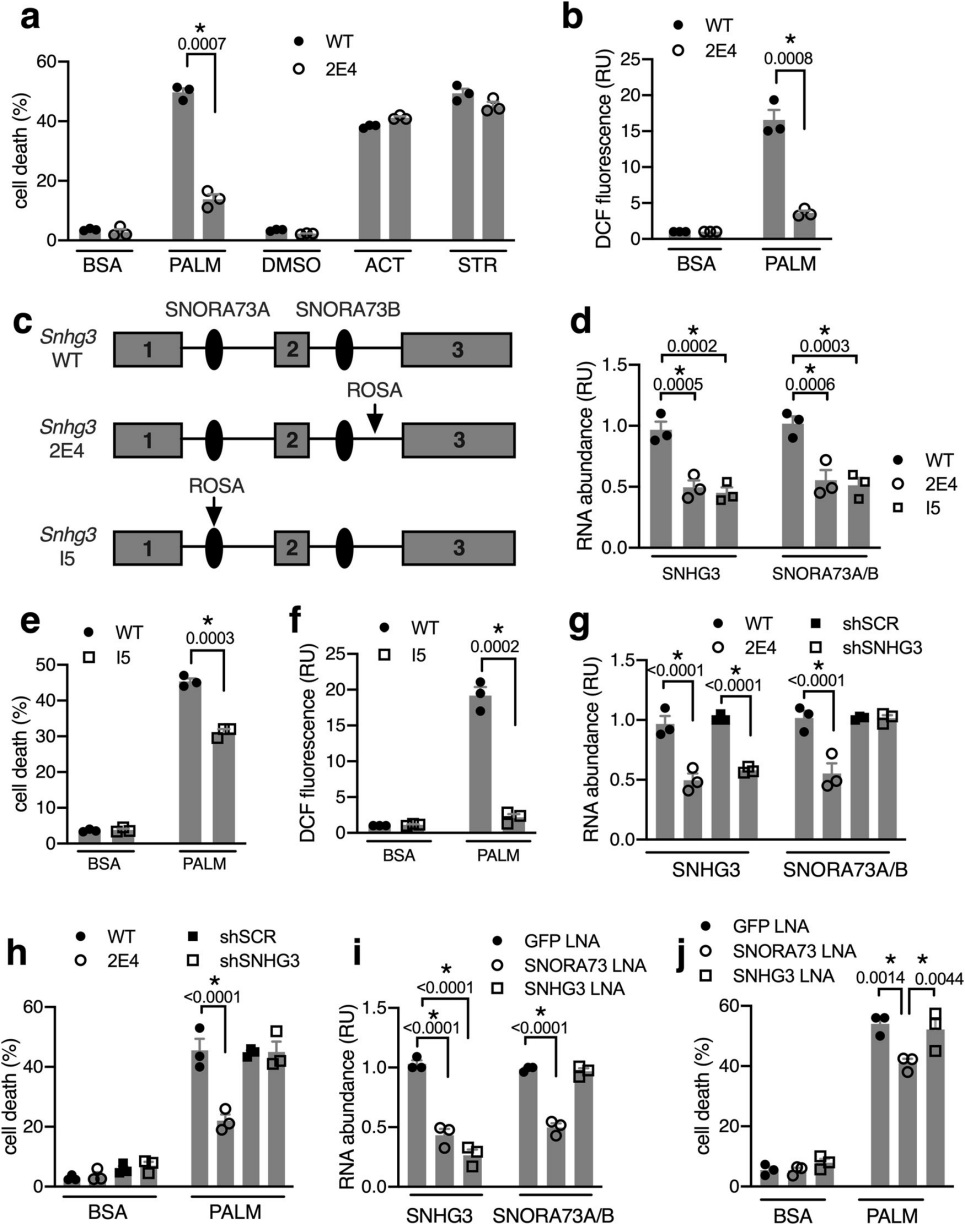
*Snhg3* mutants are resistant to lipotoxicity. **a** Cell death in CHO wild-type (WT) and mutant 2E4 cells treated with palmitate complexed to BSA (PALM) vs. BSA carrier alone, or treated with actinomycin D (ACT) or staurosporine (STR) vs. vehicle. **b** ROS by CM-H_2_DCFDA (DCF) staining in cells treated with PALM or BSA. RU, relative units. **c** *Snhg3* locus showing exons as gray rectangles, snoRNAs as black ovals, arrows for ROSAβgeo (ROSA) proviral integration sites in mutants 2E4 and I5. **d** RT-qPCR of SNHG3 lncRNA and SNORA73 relative to *Rplp0* mRNA in WT and mutant cells using qPCR primers that amplify shared regions of SNORA73A and SNORA73B. **e**, **f** PALM-induced cell death (**e**) and ROS (**f**) in WT and I5 cells. **g** RT-qPCR of SNHG3 lncRNA and SNORA73 in WT CHO and 2E4 cells, and in WT CHO cells stably expressing scrambled (shSCR, control) or SNHG3 (shSNHG3) shRNA constructs. **h** Cell death following treatment of cells in (**g**) with PALM vs. BSA. **i** RT-qPCR of SNHG3 lncRNA and SNORA73 in NIH 3T3 cells transfected with LNAs targeting GFP (control), SNHG3 lncRNA, or SNORA73. **j** Cell death following treatment of cells in (**i**) with PALM vs. BSA. Means + standard error (SE) for *n* = 3 independent experiments. *p < 0.05 for indicated comparisons (with *p*-values above brackets) by multiple unpaired *t*-tests with two-stage step-up method of Benjamini, Krieger, and Yuketieli (FDR 1%; **a**, **b**, **e**, **f**) or two-way ANOVA with Tukey’s multiple comparison test (**d**, **g**, **h**, **i**, **j**). Source data are provided as a Source data file.

Mutant 2E4 cells were isolated from a loss-of-function screen in CHO cells, designed to identify genes critical for fatty acid-induced cell death^20^. Cells were mutagenized by transduction with the ROSAβgeo retroviral promoter trap to achieve, on average, <1 integration per cell. Mutants were screened for the ability to grow in standard culture medium supplemented with 500 μM palmitic acid. Under these conditions, mutant 2E4 cells, but not wild-type (WT) CHO cells, survived. To assess the specificity of resistance to established inducers of cell death, we quantified cell death following treatment of 2E4 cells with palmitate, actinomycin D, or staurosporine. As expected, 2E4 cells were protected from palmitate-induced cell death compared to WT cells (Fig. 1a). However, sensitivity to staurosporine and actinomycin D was similar to WT, indicating that general apoptosis is intact in 2E4 mutants. Consistent with resistance to lipotoxicity, mutant 2E4 cells generated less ROS than WT cells in response to lipotoxic concentrations of palmitate (Fig. 1b). 2E4 cells are thus resistant to both lipid-induced oxidative stress and lipid-induced cell death.

Rapid amplification of cDNA ends (RACE) indicated that the retroviral promoter trap in 2E4 cells had integrated into one of two alleles of the small nucleolar RNA hosting gene 3 (*Snhg3*). This locus encodes a lncRNA, SNHG3, and two intronic box H/ACA snoRNAs, SNORA73A and SNORA73B, which are 97% identical (Fig. 1c). Among eukaryotes, SNORA73A/B are highly conserved, although the SNHG3 lncRNA sequence diverges widely^24^. The SNHG3 lncRNA is ubiquitously expressed in murine and human tissues^25^. Given that the lncRNA and snoRNAs are processed from the same pre-RNA, SNORA73A/B are also likely to be widely expressed in human and mouse tissues. Consistent with a model of haploinsufficiency at this locus, mutant 2E4 cells have 50% reduction in expression of both RNA components, SNHG3 lncRNA and SNORA73A/B, compared to WT cells (Fig. 1d). Since qPCR cannot distinguish between the highly related SNORA73A and SNORA73B, our quantification throughout refers to total SNORA73. This level of reduction in expression from the *Snhg3* locus was similar to I5 cells, an independently isolated mutant CHO line with disruption of *Snhg3*, previously shown by our group to manifest altered cellular cholesterol trafficking^26^. As with the 2E4 mutant, I5 cells are resistant to lipid-induced cell death and oxidative stress, providing independent confirmation of a role for the *Snhg3* locus in lipotoxicity (Fig. 1e, f).

To determine whether the lncRNA was the element from this locus that is required for lipotoxicity, we selectively depleted SNHG3 lncRNA in WT CHO cells and measured cell death in response to palmitate. We transduced WT cells with short hairpin constructs targeting SNHG3 lncRNA (shSNHG3) or a scrambled sequence (shSCR). Relative to shSCR cell lines, shSNHG3 cells showed 50% SNHG3 knockdown, similar to its expression in 2E4 cells, without altering abundance of SNORA73 (Fig. 1g). Upon treatment with lipotoxic concentrations of palmitate, shSNHG3 and shSCR cell lines showed no difference in cell death (Fig. 1h). This indicates that haploinsufficiency of the SNHG3 lncRNA alone is insufficient for protection from lipotoxic cell death.

Although shRNAs are highly effective for silencing RNAs in the cytoplasm, such as the SNHG3 lncRNA, an alternate approach was required to knockdown snoRNAs, which reside primarily in the nucleolus. We designed locked nucleic acid (LNA) oligomers complementary to the 3′ hairpin of SNORA73, a region that is accessible to nucleases in box H/ACA snoRNA ribonucleoproteins^27^. As controls, we designed a non-targeting LNA against GFP and an LNA to selectively deplete SNHG3 lncRNA by tiling across the exon 1-exon 2 splice junction of the mature SNHG3 lncRNA. Transfection of WT murine NIH 3T3 fibroblasts with SNORA73 LNAs resulted in 50% knockdown of SNORA73 as well as the SNHG3 lncRNA relative to the control GFP LNA, comparable to expression of these transcripts in the 2E4 mutant (Fig. 1i). This suggests that the SNORA73 LNA can target both the mature snoRNA and the *Snhg3* pre-RNA. However, transfection with the LNA targeting the splice junction of SNHG3 caused 75% knockdown of the lncRNA without affecting expression of SNORA73. Importantly, only transfection of NIH 3T3 cells with the SNORA73 LNA, but not the SNHG3 LNA, protected cells from palmitate-induced cell death (Fig. 1j). Together, our results in fibroblast lines from two different species support the hypothesis that SNORA73A/B are the elements of the *Snhg3* locus required for lipotoxicity and that the SNHG3 lncRNA is dispensable for lipotoxicity.

### *Snhg3* mutants are resistant to oxidative stress

**Fig. 2:**
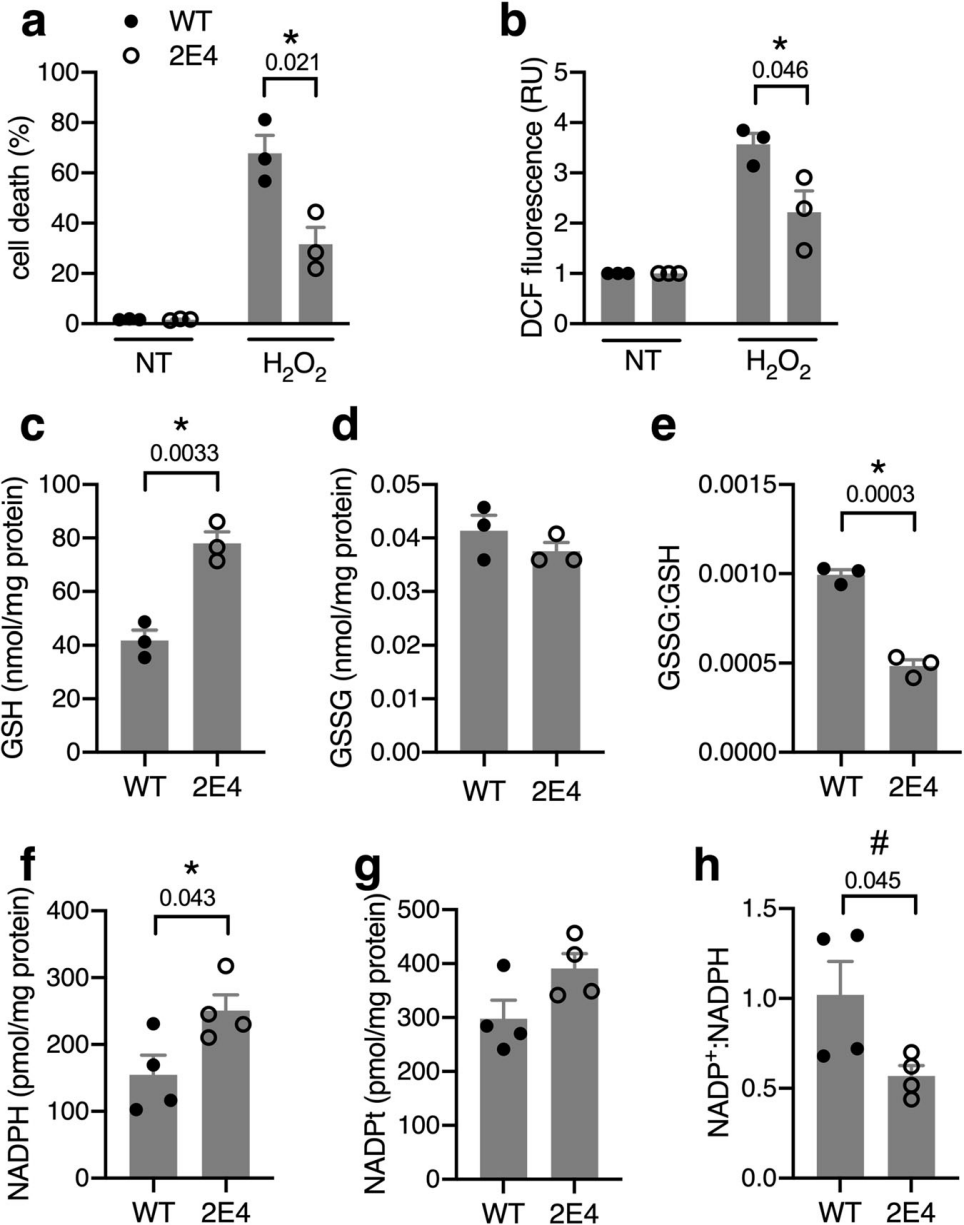
*Snhg3* mutants are resistant to oxidative stress. **a** Cell death following treatment of WT and 2E4 cells for 16 h with H_2_O_2_. NT, non-treated. **b** ROS in cells treated for 1 h with H_2_O_2_. **c**–**e** Glutathione (GSH, **c**), glutathione disulfide (GSSG, **d**), and the GSSG:GSH ratio (**e**) in WT and 2E4 cells. **f**–**h** NADPH (**f**), total NADP pool (NADPt, **g**), and NADP+:NADPH ratio (**h**) in WT and 2E4 cells. Means + SE for *n* = 3 (**a**–**e**) or 4 (**f**–**h**) independent experiments. *p < 0.05 for indicated comparisons (with *p*-values above brackets) by multiple unpaired *t*-tests with two-stage step-up method of Benjamini, Krieger, and Yuketieli (FDR 5%; **a**, **b**) or by unpaired two-tailed *t*-test (**c**, **e**, **f**). ^#^*p* < 0.05 by paired two-tailed *t*-test (**h**). Source data are provided as a Source data file.

Our finding that *Snhg3* mutants show reduced levels of palmitate-induced ROS raised the possibility that these cells are more broadly resistant to oxidative stress. Indeed, 2E4 cells treated with hydrogen peroxide show reduced cell death and ROS amplification relative to WT cells (Fig. 2a, b). Glutathione (GSH), a critical antioxidant for cell survival during oxidative stress^28^, is regenerated from its oxidized form, glutathione disulfide (GSSG), by glutathione reductases in a reaction that requires NADPH. Thus, to gauge cellular antioxidant capacity, we quantified the abundance of GSH and NADPH pools. 2E4 cells show higher GSH and a lower ratio of oxidized:reduced GSH relative to WT cells (Fig. 2c–e). They also have higher NADPH and a lower ratio of oxidized:reduced NADP(H) (Fig. 2f–h). These data suggest that reduced expression of the *Snhg3* locus leads to protection from oxidative stress by modulating the cellular redox environment.

### SNORA73 deficiency increases oxidative phosphorylation

**Fig. 3:**
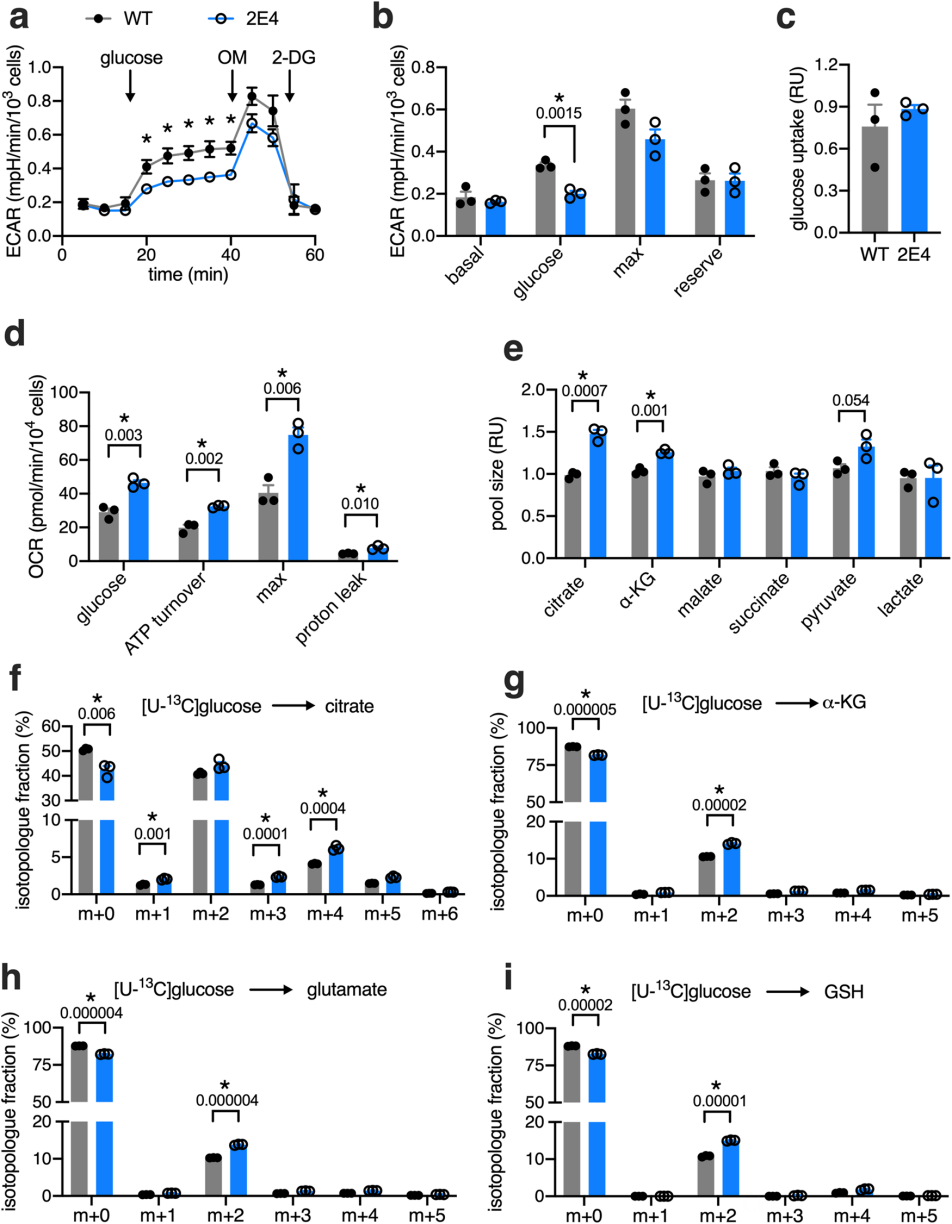
SNORA73 haploinsufficiency enhances oxidative glucose metabolism. **a**, **b** Extracellular acidification rates (ECAR) in WT and 2E4 cells in XF base medium supplemented with 2 mM glutamine (**a**). Arrows indicate injections of glucose, oligomycin (OM), and 2-deoxyglucose (2-DG). Quantification of ECAR in (**b**). **c** Glucose uptake, normalized to cellular protein content. **d** Oxygen consumption rates (OCR) in XF base medium following introduction of 5 mM glucose. Serial injections of oligomycin, FCCP, and antimycin A were used to calculate ATP turnover, maximal respiration (max), and proton leak. **e** TCA cycle intermediates (citrate, α-ketoglutarate (α-KG), malate, succinate), pyruvate, and lactate quantified by LC-MS and normalized to cellular protein content. **f**–**i** Isotopologue distribution as a fraction of total molecules of citrate (**f**), α-KG (**g**), glutamate (**h**), and GSH (**i**) by stable isotope tracing with [U-^13^C]glucose, with natural-abundance correction. Each species is identified by m + *n*, where *n* is the number of ^13^C labels incorporated and m + 0 is species without ^13^C label. Means + SE for *n* = 3 independent experiments. *p < 0.05 for indicated comparisons or for WT vs. 2E4 by two-way ANOVA with Sidak multiple comparisons test (**a**) or by multiple unpaired *t*-tests with two-stage step-up method of Benjamini, Krieger, and Yuketieli (FDR 1%; **b**–**i**). *P*-values indicated above brackets for panels (**b**) and (**d**–**i**). In panel **a**, *p*-values from left to right: 0.0456, 0.0157, 0.014, 0.0083, and 0.0124. Source data are provided as a Source data file.

Glucose flux through glycolysis and the pentose phosphate pathway (PPP) are major contributors to cellular redox balance and NADPH levels^29^. Moreover, in many tumors, a dramatic shift toward aerobic glycolysis and augmented PPP activity, a phenomenon known as the Warburg effect, contribute to increased NADPH production and oxidative stress resistance^30^. We hypothesized that these pathways might also be upregulated in 2E4 cells, as an explanation for their altered redox state. However, 2E4 cells showed significantly lower glucose-stimulated extracellular acidification rates, a proxy for rates of glycolysis, compared to WT cells, despite similar rates of glucose uptake (Fig. 3a–c). We also assessed the abundance of glycolysis metabolites by liquid chromatography-mass spectrometry (LC-MS). While there was a 20% increase in 2/3-phosphoglycerate levels in 2E4 compared to WT cells, other metabolites in the glycolysis pathway were not significantly increased (Supplementary Fig. 1a). Stable isotope tracer analysis with uniformly ^13^C-labeled ([U-^13^C]) glucose did not reveal any differences in the enrichment of glucose-6-phosphate, glyceraldehyde phosphate, 2/3-phosphoglycerate, or lactate (Supplementary Fig. 1b–e). PPP metabolites were not reliably detected in either WT or mutant CHO cells, consistent with prior tracing studies showing low PPP activity in CHO cell lines during the growth phase^31^.

Mitochondrial metabolism is another critical regulator of cellular redox balance through production of reducing equivalents, generation of GSH precursors, and neutralization of ROS. Electron transport chain integrity is also particularly critical in mounting defenses against hydrogen peroxide-induced cytotoxicity^32^. We reasoned that increased mitochondrial substrate oxidation could contribute to changes in redox balance and oxidative stress resistance. To assess whether *Snhg3* mutant cells have alterations in oxidative metabolism, we quantified oxygen consumption rates (OCR) in WT and 2E4 cells in response to glucose. Respiration on glucose was increased in 2E4 compared to WT cells (Fig. 3d). Oligomycin-sensitive respiration, proportional to ATP turnover, and FCCP-induced respiration, reflective of maximal oxidative capacity, were also higher in 2E4 cells than in WT cells. In addition, pool sizes of citrate and α-ketoglutarate were higher in 2E4 cells than WT cells (Fig. 3e). To determine whether this increased glucose oxidation represented augmented flux of glucose into the TCA cycle, we repeated metabolic tracing with [U-^13^C]glucose in WT and 2E4 cells and focused on TCA intermediates. 2E4 cells demonstrated significant increases in ^13^C-enrichment of citrate and α-ketoglutarate (Fig. 3f, g). Consistent with the notion that increased TCA metabolism augments GSH synthesis, we observed increased [U-^13^C]glucose enrichment in glutamate and GSH in 2E4 mutants relative to WT cells (Fig. 3h, i). Together, these data indicate that *Snhg3* loss-of-function rewires mitochondrial glucose metabolism toward increased oxidative phosphorylation and GSH biogenesis, resulting in an expanded GSH pool.

**Fig. 4:**
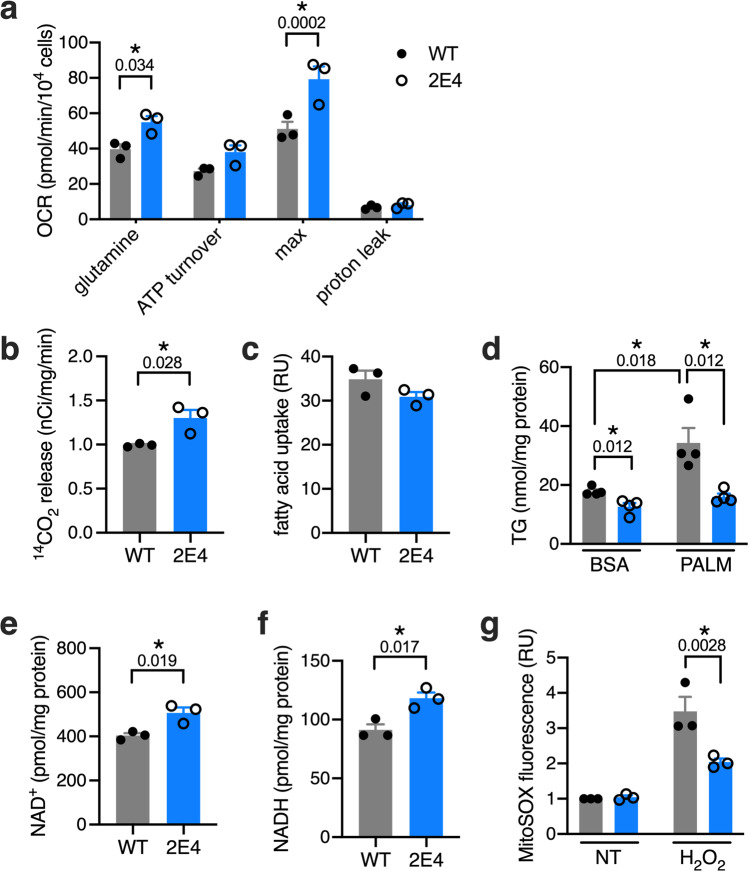
Augmented glutamine and fatty acid oxidation in 2E4 cells. **a** OCR in WT and 2E4 cells in XF base medium following introduction of glutamine. Oligomycin, FCCP, and antimycin used for calculation of ATP turnover, maximal respiration, and proton leak. **b** Fatty acid oxidation quantified by liberation of ^14^C-CO_2_ in cells incubated with [1-^14^C]palmitate. **c** BODIPY-labeled fatty acid uptake. **d** Triglyceride (TG) in cells treated with BSA or PALM for 16 h. **e**, **f** NAD+ (**e**) and NADH (**f**) in WT and 2E4 cells. **g** Mitochondrial superoxide (MitoSox) in cells treated with H_2_O_2_ for 1 h. Means + SE for *n* = 3 (**a**, **b**, **c**, **e**, **f**, **g**) or *n* = 4 (**d**) independent experiments. *p < 0.05 for indicated comparisons (with *p*-values above brackets) by two-way ANOVA with Sidak multiple comparisons test (**a**, **g**) or by unpaired two-tailed *t*-test (**b**–**f**). Source data are provided as a Source data file.

To ascertain whether this increased oxidative phosphorylation was specific to glucose, we quantified oxidation of additional substrates that feed into the TCA cycle. Consistent with a general upregulation of oxidative phosphorylation, respiration of glutamine was also significantly higher in 2E4 cells compared to WT cells (Fig. 4a). In fatty acid oxidation assays using radiolabeled palmitate, 2E4 mutants liberated significantly more ^14^CO_2_ from [1-^14^C]palmitate than WT cells, despite similar fatty acid uptake between these cell lines (Fig. 4b, c). Enhanced fatty acid oxidation was associated with lower cellular triglyceride content under both basal and lipotoxic conditions (Fig. 4d). Thus, in addition to changes in cellular redox regulation, 2E4 cells have an enhanced capacity to dispose of excess fatty acids under lipotoxic conditions. Also consistent with augmented mitochondrial oxidative capacity, we observed greater abundance of cellular NAD+ and NADH in 2E4 cells (Fig. 4e, f). NAD+ pool size is linked to mitochondrial function during oxidative stress^33^. In line with this, 2E4 cells were protected from mitochondrial ROS following challenge with H_2_O_2_ (Fig. 4g). The reprogramming of mitochondrial metabolism in 2E4 cells occurred in the absence of increases in expression of fatty acid oxidation or TCA cycle enzymes, increases in mitochondrial abundance, or altered gross mitochondrial morphology (Supplementary Fig. 2).

### Deficient rRNA processing remodels metabolism through mTOR

We next explored the mechanism through which SNORA73 deficiency leads to metabolic reprogramming. Although the canonical function of the box H/ACA class of snoRNAs is to direct isomerization of uridine to pseudouridine on nascent rRNAs, snRNAs, and tRNAs^34^, vertebrate SNORA73 and its yeast homolog snR30 are instead required for cleavage of pre-rRNAs during production of mature rRNAs^23^. However, we did not observe altered steady-state rRNA abundance in 2E4 cells, which are haploinsufficient for SNORA73 (Supplementary Fig. 3a, b). To evaluate the effects of SNORA73 deficiency on the kinetics of rRNA biosynthesis, we performed pulse-chase experiments with 5-ethynyl uridine (EU) to label newly synthesized rRNAs. During a 30 min pulse, compared to WT cells, 2E4 cells showed increased label incorporation into the 47/45S precursor species (Supplementary Fig. 3c). During a 6-h chase, the proportion of labeled 28S and 18S products steadily increased in WT cells (Supplementary Fig. 3d), whereas the proportion of labeled products failed to increase in 2E4 cells over the same time course (Supplementary Fig. 3e). These data are consistent with a model in which steady-state rRNA abundance is maintained in 2E4 cells in part through increased transcription of precursor rRNAs. The observation that labeled 28S and 18S increase over 6 h, while initial processing of the 47S rRNA precursor is essentially complete at 2 h is a function of the complex processing pathway that involves numerous endonucleolytic and exonucleolytic cleavages beyond the initial processing by SNORA73^35^. Inability to detect changes in the decay of the 47S rRNA species likely reflects the limited resolution of metabolic labeling in the setting of rapid *Xrn2*-mediated degradation of aberrantly cleaved pre-rRNAs^36^.

**Fig. 5:**
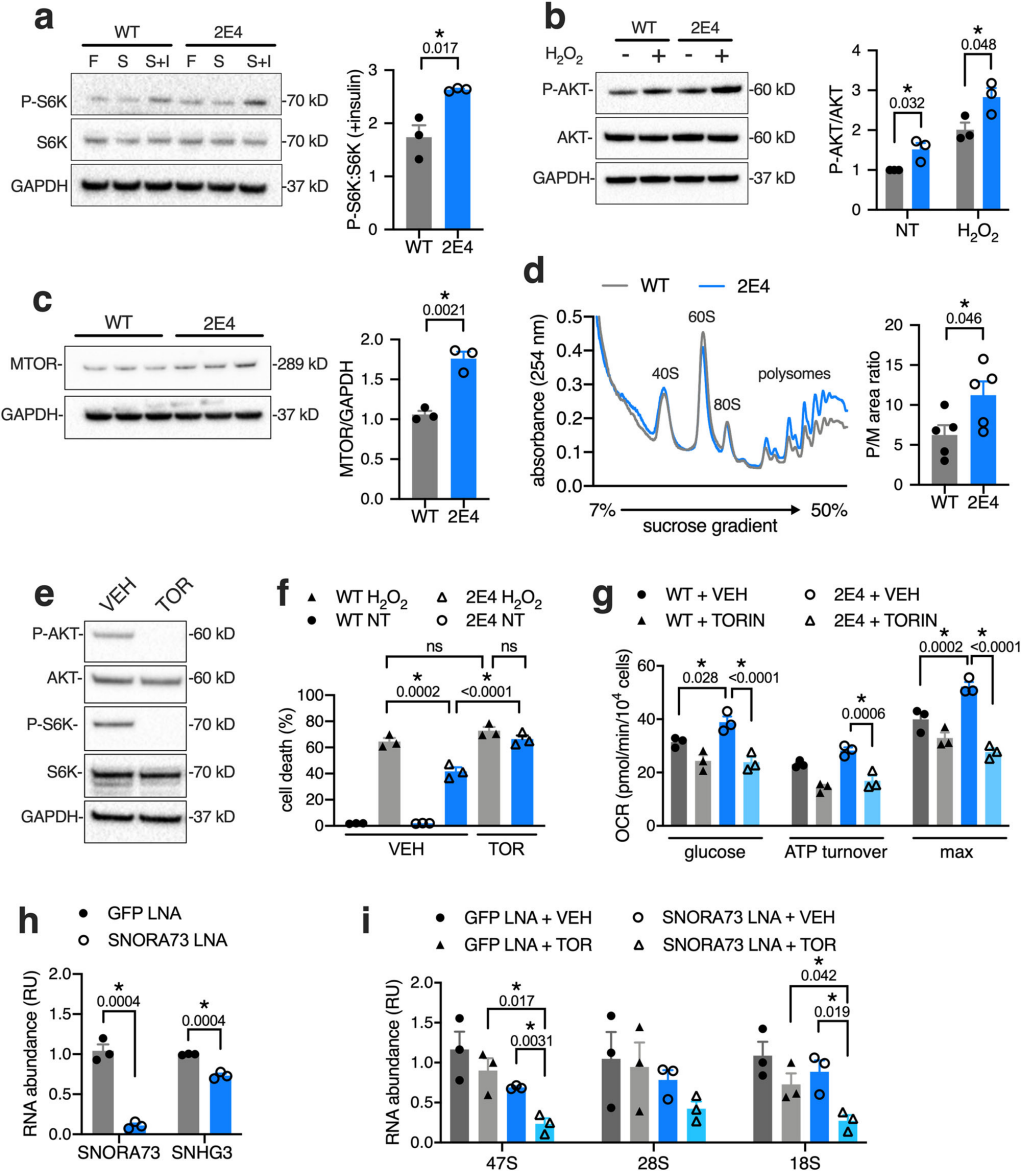
mTOR activation causes oxidative stress resistance, metabolic rewiring, and maintains rRNA production during SNORA73 deficiency. **a** Immunoblot of S6 kinase (S6K) and p-S6K (T389) in WT and 2E4 cells cultured 5% FBS medium (F), serum-free medium (S), or serum-free medium followed by treatment with insulin (S + I); quantification from independent experiments. **b** Immunoblot of AKT and p-AKT (S473) in cells treated with H_2_O_2_ with quantification. **c** Immunoblot of mTOR protein with quantification. **d** Representative polysome profiles of WT and 2E4 cells (left) with quantification of polysome to monosome peak areas (P/M ratio) for *n* = 5. **e** Representative immunoblot of P-AKT (S473), AKT, phospho-S6K (T389), S6K, and GAPDH in WT CHO cells treated for 24 h with vehicle (VEH, DMSO) or 250 nM Torin 1 (TOR). **f** Cell death in WT and 2E4 cells treated with H_2_O_2_ for 16 h in the presence of VEH or TOR. **g** OCR in XF base medium following introduction of 5 mM glucose in WT and 2E4 cells pre-treated with VEH or TOR for 24 h. **h** RT-qPCR of SNORA73 and SNHG3 lncRNA in WT cells treated with SNORA73 or GFP LNAs for 24 h. **i** RT-qPCR of 47S, 28S, 18S, and rRNAs following treatment of cells in (**h**) with TOR for 24 h. Immunoblots with quantification of independent experiments. Means + SE for *n* = 3 (**a**–**c**, **e**–**i**) or *n* = 5 (**d**). *p < 0.05 (unpaired *t*-test) for indicated comparisons (with *p*-values above brackets) by unpaired two-tailed *t*-test (**a**, **c**, **d**, **i**); by multiple unpaired *t*-tests with two-stage step-up method of Benjamini, Krieger, and Yuketieli (FDR 5%; **b**, **h**); or by one-way (**f**) or two-way (**g**) ANOVA with Tukey’s multiple comparisons test. ns, not significant. Source data are provided as a Source data file.

Ribosome biogenesis is a major energy-requiring pathway that is regulated by mTOR. In turn, defects in rRNA production activate mTOR signaling^37^. Because mTOR is also a master regulator of cellular energetics and metabolism^38^, we hypothesized that mTOR signaling might serve as a link between decreased SNORA73 and changes in cellular metabolism. Thus, we assessed signaling downstream of mTOR in WT and 2E4 cells. S6 kinase (S6K) phosphorylation at T389, a canonical target of mTORC1 signaling, was upregulated in 2E4 compared to WT cells after 16 h serum deprivation followed by administration of insulin (Fig. 5a). AKT phosphorylation at S473, mediated by mTORC2, is increased during oxidative stress where it orchestrates pro-survival responses^39,40^. We also observed increased AKT S473 phosphorylation in 2E4 relative to WT cells under both basal growth conditions and during treatment with H_2_O_2_ (Fig. 5b). In addition, expression of mTOR protein was increased in 2E4 mutants (Fig. 5c). Similarly, we observed that LNA-mediated knockdown of SNORA73 in normal human skin fibroblasts led to enhanced AKT phosphorylation, increased mTOR expression, and protection from metabolic stress (Supplementary Fig. 4). Consistent with the known function of mTOR to relieve inhibition of translational elongation and stimulate assembly of monosomes into polysomes, we observed a shift in the distribution of ribosomes toward polysomes in 2E4 cells compared to WT cells (Fig. 5d). Together, these results demonstrate that signaling pathways downstream of mTOR are activated by reduced SNORA73 expression in rodent and human cells.

To determine the contributions of mTOR activity to the phenotype of cells deficient in SNORA73, we treated cells with 250 nM Torin 1 to inhibit the mTOR signaling axis (Fig. 5e). Torin 1 abrogated both resistance to H_2_O_2_-induced cell death (Fig. 5f) and increased glucose oxidation (Fig. 5g) in 2E4 cells relative to WT cells. Torin 1 treatment also unmasked deficiencies in processing of pre-rRNA and caused significant decreases in rRNAs in cells with knockdown of SNORA73 (Fig. 5h, i), indicating that maintenance of rRNA production during SNORA73 deficiency requires mTOR signaling. Our findings support a model in which deficiency of SNORA73 impairs rRNA biogenesis. Resulting low levels of 18S and 28S rRNA activate mTOR signaling to restore rRNA levels. In the process, mTOR signaling drives metabolic rewiring and leads to lipotoxicity resistance.

### SNORA73 regulates redox and lipid homeostasis in vivo

**Fig. 6:**
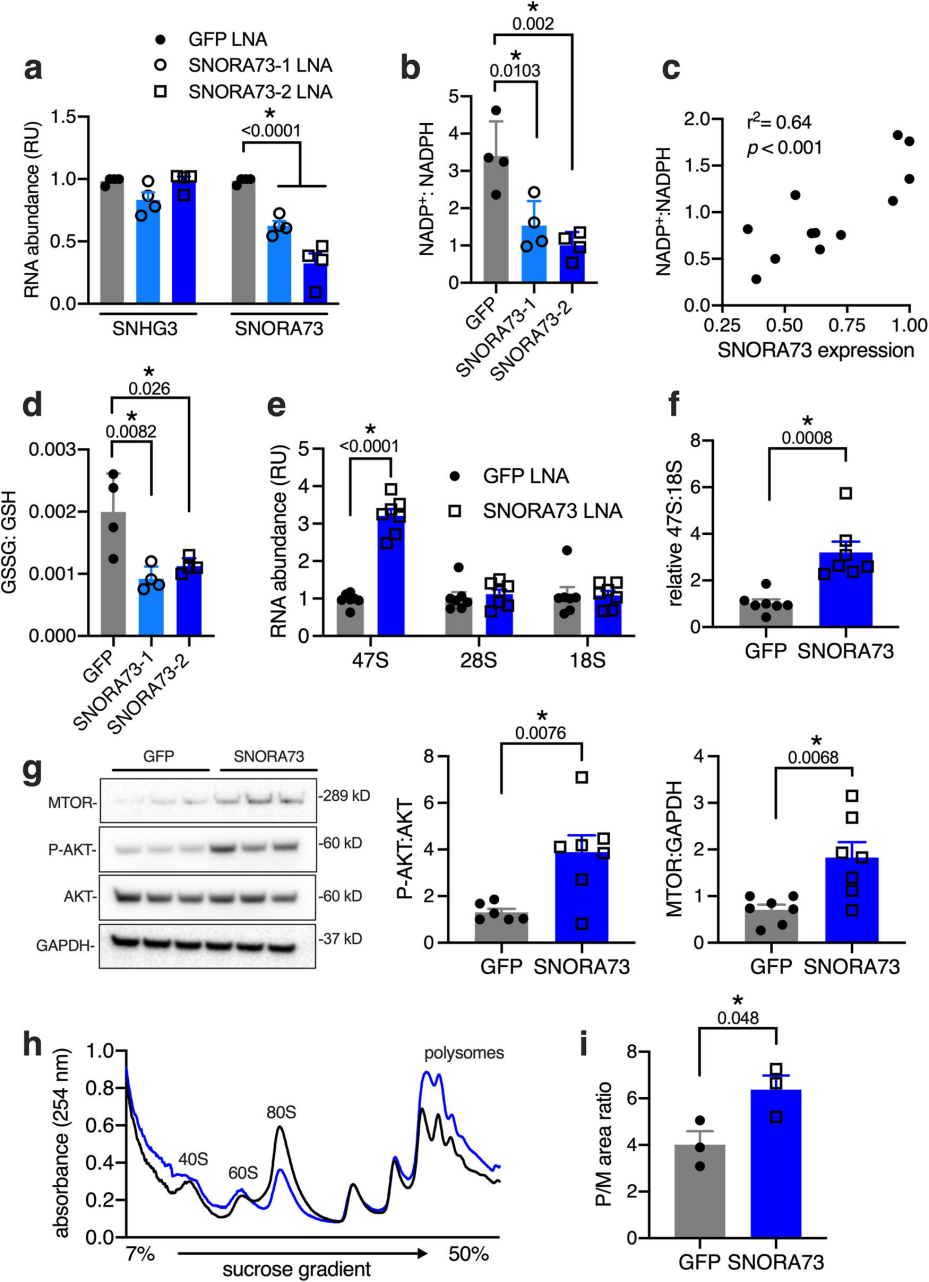
SNORA73 knockdown alters hepatic redox balance and activates mTOR. Analysis of livers of chow-fed mice treated with LNAs targeting SNORA73 (SNORA73-1, SNORA73-2) or GFP (control). **a** RT-qPCR of SNHG3 lncRNA and SNORA73 (*n* = 4 mice). **b** NADP+:NADPH (*n* = 4 mice). **c** NADP+:NADPH vs. SNORA73 expression (*n* = 12 mice). **d** GSSG:GSH (*n* = 4 mice). **e** RT-qPCR of 47S, 28S, and 18S rRNAs (*n* = 7 mice). **f**, Ratio of 47S to 18S rRNA (*n* = 7 mice). **g** Representative immunoblot of AKT, p-AKT (S473), and mTOR with quantification of P-AKT/AKT and mTOR for *n* = 7 mice (one outlier value excluded for p-AKT/AKT quantification with GFP LNA). **h**, **i** Representative polysome profiles (**h**) and quantification of polysome to monosome peak (P/M ratio, **i**) for *n* = 3 mice. Means + SE. *p < 0.05 for indicated comparisons (with *p*-values above brackets) by one-way (**b**, **d**) or two-way (**a**, **e**) ANOVA with Tukey’s multiple comparison test, or by unpaired two-tailed *t*-test (**f**, **g**, **i**). Source data are provided as a Source data file.

To extend our findings in vivo, we examined the effects of SNORA73 loss-of-function in the liver, where metabolic and oxidative stress responses are critical for organ homeostasis. We injected mice with LNAs targeting SNORA73 or GFP (control). Relative to control LNAs, SNORA73-1 and SNORA73-2 LNAs achieved 37% and 68% knockdown of SNORA73, respectively (Fig. 6a). In chow-fed animals, knockdown of SNORA73 in the liver led to higher NADPH levels, a lower NADP+:NADPH ratio, and a lower GSSG:GSH ratio, indicative of more robust antioxidant defenses (Fig. 6b–d, Supplementary Fig. 5a–d). We further examined in vivo effects of knockdown with SNORA73-2 LNA, which is more potent and does not alter expression of SNHG3 lncRNA (Fig. 6a). Knockdown of SNORA73 in the liver increased abundance of pre-rRNA, but did not impact abundance of 28S and 18S product species, leading to a higher 47S to 18S rRNA ratio (Fig. 6e, f). Consistent with our observations in SNORA73 haploinsufficient cells, SNORA73 knockdown increased AKT S473 phosphorylation and elevated mTOR expression in the livers of ad libitum-fed mice (Fig. 6g). Activation of mTOR signaling led to a concomitant shift of ribosome populations toward polysomes in the livers of mice treated with SNORA73 LNAs (Fig. 6h, i). Taken together, our findings demonstrate that loss of SNORA73 also activates mTOR signaling in vivo, and the associated increased transcription of precursor 47S rRNA species compensates to maintain wild-type levels of mature 18S and 28S rRNAs.

**Fig. 7:**
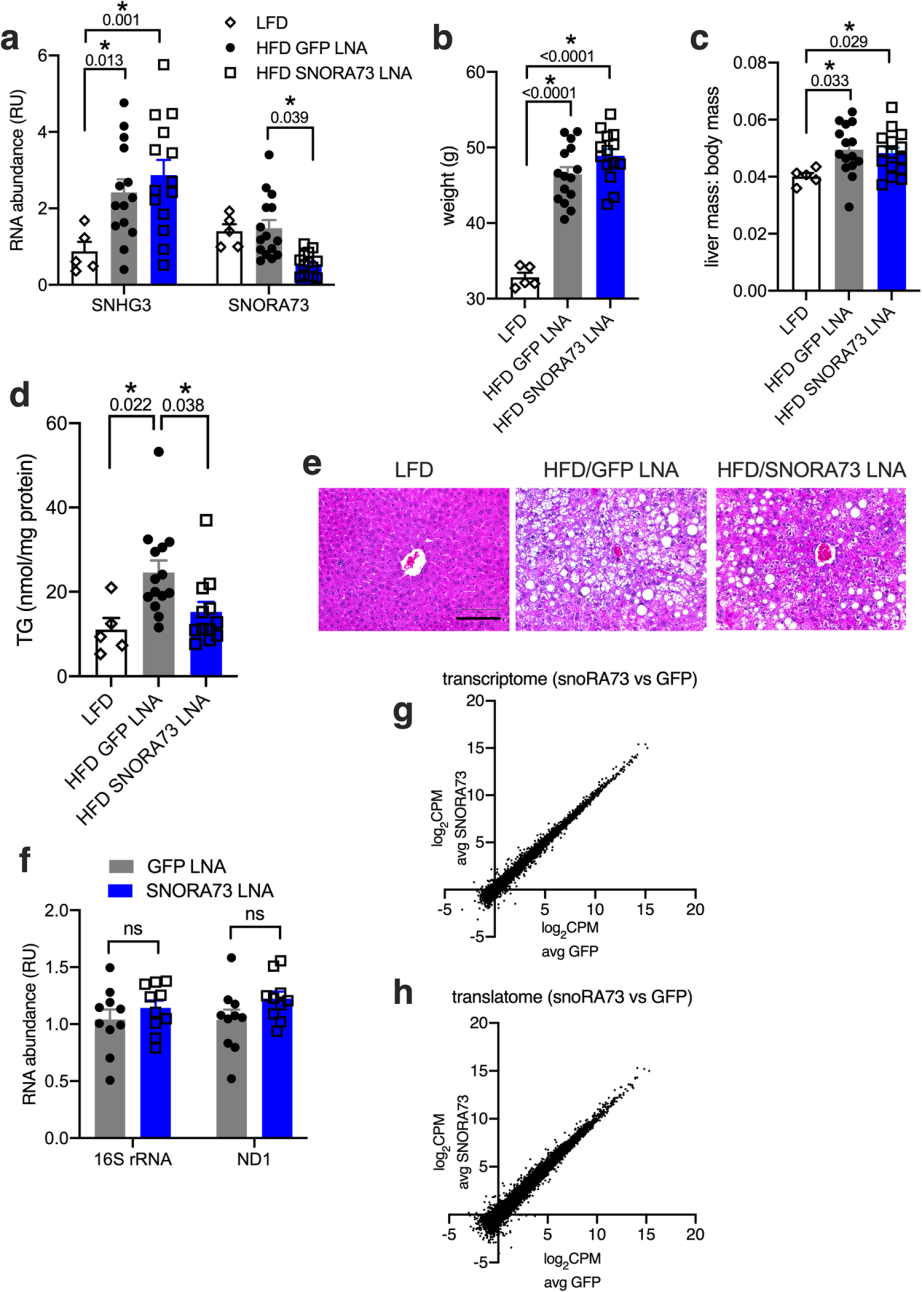
SNORA73 knockdown reduces hepatic steatosis. Mice fed a low-fat diet (LFD) or 60% high-fat diet (HFD) for 17 weeks were treated with LNAs targeting SNORA73 (SNORA73-2) or GFP (control) for the final 4 weeks of diet treatment. **a** RT-qPCR of SNHG3 lncRNA and SNORA73 in livers, **b** body weight, **c** liver mass to body mass ratio, **d** hepatic TG. **e** Representative H & E stained liver sections. Scale bar 150 µm. **f** Mitochondrial DNA (16S rRNA, mt-ND1) relative to nuclear DNA (HK2) by qPCR. **g**, **h** RNA-seq analysis of total RNA (**g**) and polysome RNA (**h**) from chow-fed animals treated with SNORA73 or GFP LNAs. For **a**–**d**, each data point derives from one mouse that received LFD (*n* = 5), HFD and GFP LNA (*n* = 15), or HFD and SNORA73 LNA (*n* = 15) with exclusion of data point outliers based on Grubbs or Rout test. For **f**, each data point derives from one mouse that received standard chow and either GFP LNA or SNORA73 LNA (*n* = 10 per group). For **g** and **h**, n = 4 mice for SNORA73 LNA and n = 4 mice for GFP LNA. Means + SE. *p < 0.05 for indicated comparisons (with *p*-values above brackets) by one-way (**b**, **d**) or two-way (**a**, **f**) ANOVA with Tukey’s (**a**) or Sidak (**f**) multiple comparison test or by unpaired two-tailed *t*-test (**c**). ns, not significant. Source data are provided as a Source data file.

To determine whether SNORA73 knockdown impacted hepatic steatosis under conditions of lipid excess, mice fed a 60% high-fat diet (HFD) for 17 weeks were treated with SNORA73 or control LNA for the final four weeks of diet intervention. Levels of SNORA73 were unchanged by HFD and LNA targeting SNORA73 achieved 64% knockdown (Fig. 7a). Expression of SNHG3 was induced by HFD, and this was unaffected by SNORA73 LNA. HFD-fed mice were obese, and knockdown of SNORA73 did not impact body weight or liver mass, both of which were increased in HFD-treated mice (Fig. 7b, c). As expected, HFD-treated mice developed hepatic steatosis (Fig. 7d, e). However, liver triglycerides were reduced by 38% in animals receiving SNORA73 LNAs, indicating that depletion of SNORA73 can reverse hepatic steatosis in the setting of lipid overload. Together, our findings demonstrate that selective knockdown of SNORA73 in vivo is sufficient to recapitulate central phenotypic aspects of *Snhg3* mutant cells.

It is well established that mTOR signaling has pleiotropic effects on mitochondrial metabolism that could contribute to increased disposal of excess lipid substrates. Mitochondrial biogenesis is unlikely the mechanism of increased mitochondrial metabolism^41^, because mitochondrial to nuclear DNA ratios were unchanged in SNORA73 knockdown versus control livers (Fig. 7f). Previous studies have shown that mTOR controls the abundance of mitochondrial oxidative function transcripts in skeletal muscle tissues and cells^42^. Thus, to comprehensively screen for changes in gene expression, we analyzed the transcriptomes of livers with SNORA73 knockdown vs. control from mice maintained on a chow diet (Fig. 7g, Supplementary Table I, *n* = 4 mice/condition). There were 74 transcriptionally upregulated genes (log_2_ ≥ 0.50, FDR < 0.05). In pathway analyses, genes related to transmembrane receptor protein phosphatase activity, cell adhesion, and cell–cell junctions were over-represented. There were 20 transcriptionally downregulated genes (log_2 _< 0, FDR < 0.05). Pathways related to cytokine responses, response to hormone, and alpha-actinin binding were over-represented in this group. Among genes related to mitochondrial metabolism, we observed upregulation of mt-ND6 (1.97-fold) and downregulation of PPARγ (−1.67-fold). Expression of mt-ND6 is critical for mitochondrial complex IV activity in skeletal muscle^43^ and disruption of hepatic PPARγ improves hepatic steatosis in ob/ob mice^44^, prompting us to consider whether changes in these mRNAs might underlie enhanced mitochondrial metabolism and decreased steatosis. Nonetheless, the changes we observed in abundance of these protein-coding transcripts are of uncertain significance in the absence of corresponding changes for these genes in the translatome (see below). Abundance of mRNAs for other metabolic transcription factors, enzymes, and oxidative phosphorylation complexes was not significantly changed.

mTOR activation can also act downstream of transcription to increase translation of mRNAs that promote mitochondrial oxidative function^45^. To address the possibility that mTOR activation of translation may drive enhanced mitochondrial metabolism, independent of transcriptional regulation, we sequenced polysomal RNAs from the livers of SNORA73 and control LNA-treated mice (Fig. 7h, Supplementary Table I). There were 30 genes whose translation was upregulated (log_2_ ≥ 0.5 FDR < 0.05), and in pathway analyses, genes related to transmembrane receptor protein phosphatase activity and cell adhesion were enriched. Translation was downregulated (log_2_ < 0; FDR < 0.05) for only 3 genes (pathway analysis not performed). Among genes related to mitochondrial metabolism, none were significantly upregulated in the polysomes (FDR < 0.05). While we observed a trend for reduced translation of PPARγ in SNORA73 LNA-treated livers (−1.99-fold), this did not reach statistical significance (FDR > 0.1). Thus, in contrast to findings in cultured breast cancer and embryonic fibroblast cell lines, we did not find broad genome-wide changes in gene expression that are likely to underlie increases in mitochondrial oxidative function. This suggests that mTOR signaling in our model impacts metabolism downstream of gene expression, through signaling nodes such as AKT that converge on the mitochondria^46,47^.

### Knockdown of SNORA73 ameliorates diet-induced lipotoxicity

**Fig. 8:**
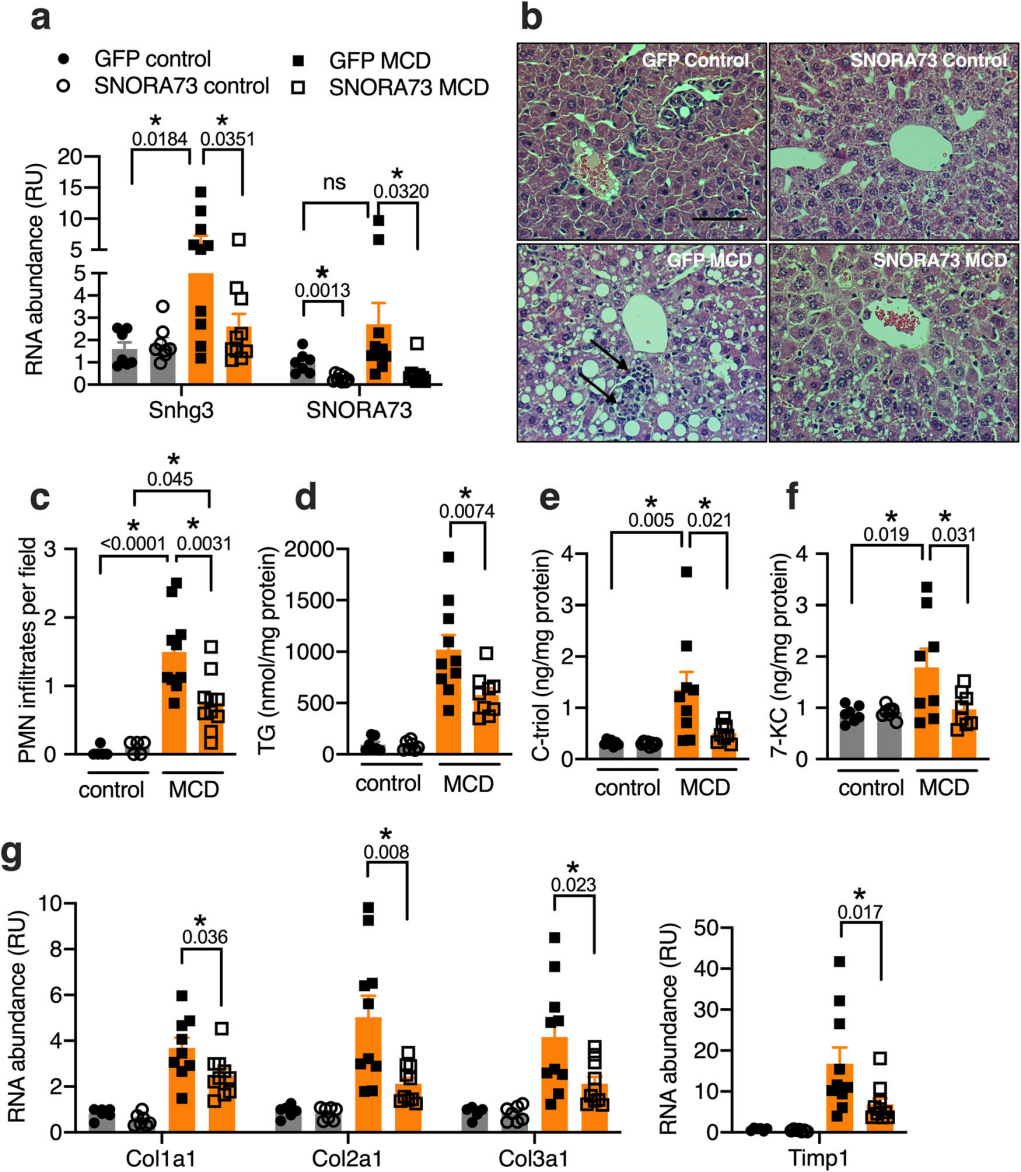
SNORA73 knockdown ameliorates steatohepatitis. Mice were treated with control (GFP) or SNORA73 (SNORA73-2) LNAs during feeding with control or MCD diets for 3 weeks. **a** RT-qPCR of SNHG3 and SNORA73. **b** Representative hematoxylin/eosin-stained liver tissue. Arrows indicate polymorphonuclear (PMN) infiltrates. Scale bar, 50 µm. **c** Quantification of PMN infiltrates in (**b**). **d** Liver TG. **e**, **f** LC/MS-MS quantification of liver cholestane-3β, 5α, 6β-triol (C-triol, **e**), and 7-ketocholesterol (7-KC, **f**). **g** RT-qPCR of transcripts associated with fibrogenic response. Col1a1, collagen type 1 alpha 1; Col2a1, collagen type 2 alpha 1; Col3a1, collagen 3 alpha 1; Timp1, tissue inhibitor matrix metalloprotease 1. Each data point derives from one mouse that received GFP LNA and control (*n* = 7) or MCD (*n* = 10) diet, or SNORA73 LNA and control (*n* = 8) or MCD (*N* = 10) diet. In **c**, 5 representative control diet samples were quantified. In **e** and **f**, samples were analyzed from GFP LNA and control (*n* = 7) or MCD (*n* = 9) diet, or SNORA73 LNA and control (*n* = 8) or MCD (*N* = 9) diet. In **g**, RNA was not available for one GFP LNA mouse (*n* = 6). Data point outliers were excluded based on Grubbs or Rout test. Means + SE. *p < 0.05 for indicated comparisons (with *p*-values above brackets) by unpaired two-tailed *t*-test (**a**, **g**), or by one-way ANOVA with Tukey’s multiple comparison test (**c**–**f**). Source data are provided as a Source data file.

In cultured cells, knockdown or haploinsufficiency of SNORA73 protects against lipid-induced oxidative stress and cell death. While the 60% HFD-fed model develops steatosis, this is well-tolerated up to 19 weeks, without robust inflammation or progression to fibrosis^48^. To test whether knockdown of SNORA73 protects against lipid-induced liver injury, we examined the effects of SNORA73 knockdown in mice fed a methionine- and choline-deficient diet (MCD). We administered LNAs targeting SNORA73 or control LNAs targeting GFP to C57BL6/J male mice and placed these animals on the MCD for 3 weeks. LNAs achieved effective knockdown of SNORA73 in mice on both control and MCD diets (Fig. 8a). Relative to animals receiving control diet, expression of the SNHG3 lncRNA, but not SNORA73, was significantly induced by the MCD diet, consistent with our findings in the 60% HFD. MCD diet feeding caused similar weight loss and elevations of plasma transaminases in animals that received control and SNORA73 LNAs (Supplementary Fig. 6a, b). Liver sections of MCD-fed control knockdown animals showed marked steatosis and inflammatory mononuclear and polymorphonuclear (PMN) infiltrates (Fig. 8b). In comparison, liver tissue of MCD-fed animals with SNORA73 knockdown showed less lipid accumulation and fewer PMNs (Fig. 8c). Overall F4/80 staining intensity was increased in animals on the MCD diet but was not affected by SNORA73 knockdown (Supplementary Fig. 6c). However, the abundance of crown-like structures of F4/80 positive cells surrounding dying hepatocytes was decreased with SNORA73 knockdown compared to control. Biochemical analyses confirmed that knockdown of SNORA73 significantly reduced hepatic triglyceride content (Fig. 8d). Furthermore, cholestane-3β, 5α, 6β-triol, and 7-ketocholesterol, robust lipid markers of tissue oxidative stress^49^, were diminished by SNORA73 knockdown in animals on the MCD diet (Fig. 8e, f). Treatment with the MCD diet for this short duration produced minimal fibrosis (Supplementary Fig. 6d). However, transcripts associated with stellate cell activation and fibrosis, including collagens and matrix remodeling proteins, were significantly increased in MCD-fed animals that received control LNAs relative to MCD-fed animals receiving SNORA73 LNAs (Fig. 8g). The observations that knockdown of SNORA73 in vivo reduced steatosis and markers of tissue damage are consistent with a model in which depletion of SNORA73 protects against lipotoxicity.

## Discussion

In this study to identify regulators of the response to lipid-induced metabolic stress, we establish links between snoRNAs, oxidative metabolism, and cellular redox regulation. We show that cells deficient in SNORA73—generated by promoter trap mutagenesis and independently confirmed by knockdown—are protected from lipotoxic and oxidative stress. This resistance is associated with increased levels of the major antioxidant, GSH, with a shift in the GSSG:GSH ratio away from oxidation and increased cellular levels of NADPH and NADH. Mutant cells haploinsufficient for SNORA73 have increased mitochondrial oxidative metabolism that drives GSH biogenesis. We demonstrate that the critical link between loss of SNORA73 and this metabolic rewiring is altered kinetics of rRNA production that leads to enhanced mTOR signaling. In cells deficient in SNORA73, mTOR signaling compensates to maintain cellular rRNA levels, and in the process drives metabolic rewiring that confers resistance to metabolic stress. We extend these findings in vivo in a setting in which lipotoxicity plays a key role in pathogenesis. Our studies show that selective knockdown of SNORA73 in mice confers protection from HFD-induced steatosis, and from steatosis, inflammation, and oxidative stress induced by the MCD diet. Thus, our study provides a link between snoRNA loss of function and amelioration of lipotoxicity in vivo.

Rewiring of mitochondrial oxidative metabolism in 2E4 cells may protect against lipotoxicity in several ways. First, similar to observations in cells treated with the AMPK activator AICAR^7^, increased fatty acid oxidation enables 2E4 cells to dispose of excess fatty acid substrates through β-oxidation. Diminished cellular triglyceride stores in 2E4 cells under lipotoxic conditions are a reflection of this enhanced utilization. Second, TCA metabolism fuels mitochondrial ROS-neutralizing enzymes, including superoxide dismutases, GSH peroxidases, and thioredoxins through the generation of α-ketoglutarate, a precursor for GSH synthesis, and through NADPH-producing enzymes including malic enzyme, isocitrate dehydrogenase 2, glutamine dehydrogenase, and NADP(H) transhydrogenase^50,51^. The importance of these mitochondrial antioxidant defenses is underscored by the observations that mitochondrial dysfunction is associated with insulin resistance, NAFLD, and development of type 2 diabetes^52–54^. In the present study, we show that the 2E4 mutant has an expanded pool of α-ketoglutarate with greater contribution of carbons from labeled glucose into this intermediate, findings indicative of enhanced TCA flux. In addition, 2E4 cells have enhanced production of GSH and a diminished GSSG/GSH ratio. Although fatty acid and glucose metabolism are often regulated reciprocally, 2E4 cells have upregulation of oxidative metabolism of both fatty acids and glucose, consistent with previous findings in a number of physiological stresses^55^.

Both metabolic reprogramming and protection from oxidative stress in 2E4 cells require activation of mTOR signaling pathways. In the setting of SNORA73 deficiency, mTOR signaling is also required for maintenance of cellular rRNA content. It is well appreciated that signaling via mTOR regulates rRNA biogenesis. In the presence of mitogenic stimuli, mTORC1 associates with promoters of rRNA genes and recruits RNA polymerase I to increase transcription of new rRNA^56^. At the same time, mTORC1 activates numerous rRNA processing events that lead to mature 18S, 28S, and 5.8S rRNA^57^. Our findings in the 2E4 mutant are consistent with a model in which communication between mTOR signaling pathways and rRNA synthesis is bidirectional, such that 2E4 cells sense low levels of 18S and 28S rRNA and provide feedback to activate mTOR signaling pathways in a compensatory manner^37,58^.

Our study provides mechanistic insights regarding SNORA73 and lipid metabolism in vivo. Both the 60% HFD and the MCD diet produce robust steatosis, and the MCD diet induces rapid steatohepatitis with oxidative stress that has been used as a model of NAFLD^48,59,60^. Here, we show that deficiency of SNORA73 reduces lipid accumulation in both models and limits tissue damage with MCD. We used a 3-week study endpoint in the MCD model to focus on the early consequences of hepatic steatosis and to avoid the increased mortality associated with longer MCD treatments. While we cannot fully assess the function of SNORA73 in development of fibrosis due to the short duration of our MCD study, SNORA73 knockdown blunted MCD diet-induced expression of stellate cell activation markers. We acknowledge limitations of these models. The 60% HFD induces weight gain, hyperlipidemia, and insulin resistance, and leads to hepatic steatosis in C57BL6J mice over weeks^48^. However, there is little evidence for multifocal inflammatory infiltrates, fibrosis, or hepatocellular damage, unless exposures are carried out for extended periods (~50 weeks), and there is no evidence for hepatocellular ballooning. Thus, while this model recapitulated many of the systemic features of early NAFLD, it does not progress to NASH. The MCD diet exhibits many features of the intrahepatic pathology of human NAFLD including steatosis with lobular and periportal inflammation, oxidative stress, and hepatocellular injury that progresses to fibrosis^59^. However, substantial weight loss and insulin sensitivity are features of this model that do not reflect systemic features of NAFLD. Despite these limitations, we chose the HFD and MCD models and the short duration of studies, because our goal was to evaluate the effects of SNORA73 deficiency on steatosis and early markers of lipotoxic injury (e.g., oxidative stress and inflammation). Moreover, we sought a time frame suitable for achieving SNORA73 deficiency via LNA-mediated knockdown, as several attempts by our group to use CRISPR/Cas9 to insert LoxP sites into this locus have been unsuccessful. An important extension of this work will be to move to models such as the fructose, palmitate, cholesterol (FPC) diet, which better recapitulates both hepatocellular and systemic features of NASH^61^ and to achieve stable haploinsufficiency of the intronic snoRNAs (e.g., by knockin of a locus lacking the snoRNAs^62^).

Our data links snoRNAs from the *Snhg3* locus to the progression of NAFLD. To our knowledge, there are no prior reports linking SNORA73 abundance to human NAFLD and no known variants, SNP, or eQTL associations for SNORA73. Previous studies have correlated levels of more than 20 lncRNAs with NAFLD by virtue of up or downregulation of the lncRNA in mouse models of the disease or in samples from affected human subjects, but notably the SNHG3 lncRNA is not among this list^63^. Our finding that abundance of SNHG3 is increased following 1*7* weeks of HFD, along with the presence of multiple metabolically regulated transcriptional response elements upstream of the promotor (e.g., CREB, PPARα, SREBP), suggest that the *Snhg3* locus could be subject to transcriptional regulation in response to nutrients. However, HFD has no impact on levels of SNORA73, implicating distinct post-transcriptional mechanisms for regulation of steady-state abundance of the two classes of RNAs produced from the same precursor RNA. Studies of hepatocellular carcinoma tumor tissues have reported increased abundance of RNA from the *Snhg3* locus compared to normal liver tissue that predicts worse clinical outcomes^64–66^, and gain- and loss- of an SNHG3 cDNA in hepatoma cells impacts proliferation and expression of genes involved in epithelial–mesenchymal transition^65–67^. However, these studies examined tumors outside the context of NAFLD, and the methods employed neither quantified nor targeted SNORA73.

Disruption of the *Snhg3* locus was previously reported by our group in the I5 mutant, which was isolated in a screen for mutants defective in intracellular cholesterol trafficking^26^. While both the I5 mutant and the 2E4 mutant in the present study are protected against lipotoxicity, resistance is stronger in 2E4 cells, despite similar decreases in SNORA73 as detected by a qPCR assay that quantifies total SNORA73. In I5 cells, promotor trap integration occurred within SNORA73A sequences in the first intron, whereas promotor trap integration in 2E4 cells occurred within intron 2 that contains SNORA73B. Subtle differences in phenotypes of the cells may relate to the functional differences between the two SNORAs. Nonetheless, our previous studies established that haploinsufficiency of SNORA73 is associated with remodeling of mitochondria-associated ER membranes (MAM), with evidence for an important role of the mRNA encoding HUMMR, an outer mitochondrial membrane adapter whose expression is regulated by SNORA73. In the present study, we show that haploinsufficiency of SNORA73 upregulates mTOR signaling to maintain rRNA levels and drives mitochondrial metabolism. mTOR signaling has been shown to be required for MAM formation, which is important for mitochondrial glucose and lipid metabolism^46^.

Our genetic screen identified a role for SNORA73 in modulating the cellular response to lipotoxic and oxidative stress. The promoter trap mutagenesis strategy we used enabled disruption of noncoding elements that are not typically targeted in standard genome-wide shRNA or CRISPR screens and may have been facilitated by the propensity of snoRNA hosting loci to serve as sites for integration of proviruses and other mobile genetic elements^68^. Demonstration that loss of SNORA73 leads to metabolic rewiring and abrogates lipotoxicity in vitro and in vivo reveals a way in which snoRNAs regulate cellular metabolism and metabolic stress. Our findings raise the possibilities that variation in snoRNA abundance or function could underlie metabolic phenotypic variability in humans and that these noncoding RNAs could serve as therapeutic targets for metabolic diseases.

## Methods

Further information and requests for resources should be addressed to the Lead Contact, Jean Schaffer (jean.schaffer@joslin.harvard.edu).

### Mice

All experimental procedures were approved by the Washington University and Joslin Diabetes Center Animal Studies Committees and conducted in accordance with the Public Health Service Policy for the Humane Care and Use of Laboratory Animals.

### Cell culture

CHO-K1 cells (ATCC CCL-61) and CHO-derived cell lines were cultured in a 1:1 mixture of Dulbecco’s modified Eagle’s medium and Ham’s F-12 nutrient medium (Sigma N6658) supplemented with 5% fetal bovine serum (Sigma F2442), 2 mM L-glutamine, and 1 mM sodium pyruvate. NIH 3T3 cells (ATCC CRL-1658) were maintained in DMEM with 10% fetal calf serum and 2 mM glutamine. For lipotoxicity experiments, growth medium was supplemented with 500 μM palmitate (Nu-Chek Prep) complexed to BSA (Sigma A8806) at a 2:1 molar ratio, as described previously^14^. Normal human skin fibroblasts (NSF, ATCC CRL-1474) were grown in DMEM supplemented with 10% inactivated fetal bovine serum.

### Chow diet study

Male C57BL/6J mice were obtained from the Jackson Laboratory (JAX 000664). Mice were housed with a 12 h:12 h light:dark cycle and fed standard chow ad libitum. For in vivo knockdown experiments, locked nucleic acids (LNA, Qiagen) targeting SNORA73 or GFP (control) were injected intraperitoneally into 8-week-old mice at a dose of 3 mg/kg every other day for 3 injections (see Supplementary Table 2). Animals were euthanized 2 days after the final injection for analyses.

### High-fat diet study

C57BL/6J male mice fed ad libitum a 60% high-fat diet (HFD, Research Diets D12492) or low-fat diet (LFD, Research Diets D12450B) beginning at 6 weeks of age. Beginning at 19 weeks, animals on HFD were injected intraperitoneally with U17 snoRNA or control LNAs at a dose of 3 mg/kg every other day for 3 injections and once weekly thereafter, while being maintained on study diet. Animals were euthanized for tissue analyses at 23 weeks, 1 week after final injection.

### Methionine-choline-deficient (MCD) diet study

Seven-week-old C57BL/6J male mice were injected intraperitoneally with SNORA73 or control LNAs (3 mg/kg every other day for 3 injections, once weekly thereafter for 3 weeks). Following the first week of LNA treatment, animals were placed on MCD diet (MP Biomedicals 960439) or matched control diet (MP Biomedicals 960441) for 3 weeks. Animals were euthanized one week after the final LNA injection for analyses. Livers were fixed in neutral buffered formalin and processed for immunohistochemistry. Plasma AST and ALT activity was quantified using the UV-kinetic method (Teco Diagnostics, AST: A559, ALT: A524).

### Genetic screen

The loss-of-function genetic screen in CHO-K1 cells to isolate lipotoxicity-resistant mutants was described previously^69^. Genes disrupted at the site of retroviral insertion were identified by 5′ RACE using an oligonucleotide tag and ROSAβgeo sequences. 5′ RACE products were TA-cloned, sequenced, and analyzed by NCBI BLASTN 2.2.16 [Mar-25-2007].

### In vitro *Snhg3* knockdown

LNAs (Qiagen) were designed to target SNORA73 and the SNHG3 lncRNA, or GFP as a control. pGFP-C-shLENTI lentiviral shRNA constructs (Origene TR30023) were designed to target hamster SNHG3 lncRNA or GFP as a control. See Supplementary Table 2. For LNA knockdown experiments in NIH 3T3 cells, 4.0 × 10^4^ cells were seeded per well in a 6-well dish and transfected the following day with 25 nM LNA using Lipofectamine 3000 (ThermoFisher L3000015). SNHG3 and SNORA73 expression and palmitate-induced cell death were analyzed 48 h following transfection. For LNA knockdown in CHO cells, 2.5 × 10^5^ cells were plated in 6-cm dishes and transfected the next day with 25 nM LNA using Lipofectamine 3000. For LNA knockdown in primary human fibroblasts, 5.0 × 10^4^ cells were plated in 6-well plates, or 1.5 × 10^5^ cells were seeded in 6-cm dishes. Cells were transfected with 25 nM LNA the following day using Lipofectamine 3000. For shRNA transduction experiments, virus was harvested from HEK293T cells (ATCC CRL-3216) that were transfected using Lipofectamine 3000 with shRNA constructs and Mission helper plasmids (Sigma SHP001). 5.0 × 10^5^ CHO cells were seeded in 6-cm dishes, grown for 24 h, and transduced with shSCR or shSNHG3 lentiviral particles. After expansion of the population, the top 20 percent of cells were flow-sorted and maintained as the transduced population.

### Cell death assays

WT and mutant CHO cells were treated for 48 h with 500 µM palmitate complexed to BSA (PALM) vs. BSA carrier alone, or treated for 24 h with 2 µM actinomycin D or 80 nM staurosporine vs. DMSO vehicle, or treated for 16 h with 2 mM H_2_O_2_. NIH 3T3 cells transfected with LNAs were treated for 24 h with 400 µM PALM vs. BSA. NSFs transfected with LNAs were treated for 48 h with 1 mM PALM vs. BSA or with 750 µM H_2_O_2_ for 16 h. Cell death was quantified by flow cytometric analysis (10^4^ cells/sample) of annexin V-EGFP (BioVision 1004) and propidium iodide (ThermoFisher 1304MP) staining using a BD LSRFortessa and BD FACSDiva software and using FlowJo v10.6.1 for analyses (Supplementary Fig. 7). Annexin V-EGFP and/or propidium iodide positive cells were considered dead.

### Detection of ROS

Following treatment of WT and mutant CHO cells with PALM or BSA for 16 h, or with 2 mM H_2_O_2_ for 1 h, cells were rinsed with PBS and incubated in PBS containing 0.5 mM MgCl_2_, 0.92 mM CaCl_2_, and 3 μM CM-H_2_DCFDA (ThermoFisher C6827) for 1 h at 37 °C or 2.5 μM MitoSox Red (ThermoFisher M36008) for 20 min at 37 °C. Cells were trypsinized and mean fluorescence was determined by flow cytometric analysis of 10^4^ cells/sample.

### Live cell imaging

CHO and 2E4 cells were transduced with retroviral particles containing plasmids encoding roGFP1 tagged with a mitochondrial localization sequence^70^. 1 × 10^5^ cells were seeded in 35 mm glass-bottom culture dishes and visualized using a Zeiss LSM 880 Airyscan microscope.

### Cellular respiration assays

Oxygen consumption rates (OCR) were measured using a Seahorse XF24 analyzer (Agilent). 4.0 × 10^4^ cells were seeded per well in Seahorse culture plates (Agilent 100850) and incubated overnight in growth medium. Cells were incubated in XF base medium (Agilent 102353) for 1 h at 37 °C in a CO_2_-free incubator. OCR was determined after injection of 5 mM glucose or 2 mM glutamine, and following serial additions of 1 μM oligomycin (Sigma 75351), 2 μM fluorocarbonyl cyanide phenylhydrazone (FCCP, Sigma C2920), and 2 μM antimycin A (Sigma A8674) in order to calculate ATP turnover, maximal respiration, and proton leak, respectively. After the assay, cells were rinsed with PBS, trypsinized, and quantified by trypan blue staining. For experiments measuring OCR with mTOR inhibitors, 2.0 × 10^4^ cells were seeded per well in Seahorse culture plates. The following day, cells were treated with DMSO or Torin 1 (250 nM, Tocris 4247) for 24 h prior to measurement of OCR as described above.

### Measurement of NAD(H) and NADP(H)

Nicotinamide adenine nucleotides were quantified in CHO and 2E4 cells (1 × 10^6^ cells) and liver tissue (10 mg) using an NAD + /NADH Assay Kit (Abcam ab65348) and an NADP/NADPH Assay Kit (Abcam ab65349) according to manufacturer instructions.

### LC-MS metabolite analysis

1 × 10^6^ CHO and 2E4 cells were seeded in 6-cm dishes and incubated overnight in growth medium. Cells were rinsed in PBS and incubated in growth medium (DMEM-low glucose (Sigma D6046), 5% FBS, 2 mM glutamine) containing 15 mM [U-^13^C]glucose (Cambridge Isotope Laboratories CLM-1396) for 6 h. Polar metabolites were extracted from cellular and culture medium samples with 80% methanol in water (v/v). Samples were analyzed on a Dionex UltiMate 3000 UHPLC coupled to a Thermo Scientific Q Exactive Plus Orbitrap. 3 µL of the reconstituted cell extracts and 2 µL of media samples (1:100 and 1:1000 dilution) were injected onto a SeQuant ZIC-pHILIC column (2.1 × 100 mm, 5 µm) at 40 °C. Mobile phase A was 95% water, 5% acetonitrile (v/v) with 20 mM ammonium bicarbonate, 0.1% ammonium hydroxide, and 4 µM medronic acid. Mobile phase B was 95% acetonitrile, 5% water (v/v). The flow rate was set to 0.25 mL/min and the following gradient was applied: 0–1 min, 90% B; 1–14 min, 90–25% B; 14–15.5 min, 25% B; 15.5–18 min, 25–90% B; 18–31 min, 90% B. From 19.5–27.5 min, the flow rate was increased to 0.4 mL/min for faster equilibration. Electrospray ionization (ESI) was used in negative polarity with 2.8 kV spray voltage. High-resolution mass spectrometry data were acquired in full scan mode from 65 to 900 *m*/*z* with 140,000 resolution and an automatic gain control (AGC) target of 1e6. The data were analyzed using Skyline 4.2^71^ and natural isotope abundance correction was performed with AccuCor^72^. For glucose uptake, glucose depletion from the culture medium was determined by LC-MS over 6 h and used to calculate relative glucose uptake, normalized to cellular protein content.

### Glutathione quantification

GSH and GSSG were extracted from 1 × 10^6^ cells or 5 mg liver tissue with 50% acetonitrile in water (v/v) spiked with 500 ng internal standards (GSH-d5 (Santa Cruz sc-489493) and ^13^C^15^N-GSSG (Toronto Research Chemicals G597972)) and derivatized with 310 mM *N*-methylmaleimide. Samples were analyzed using a 20AD Shimadzu HPLC system coupled to an Applied Biosystems API 4000 tandem mass spectrometer. Samples (3 µL) were injected onto a ThermoFisher Hypercarb column (3 μm, 150 mm × 3 mm). Mobile phase A was 1% formic acid in water and mobile phase B was 1% formic acid in 1:1 acetonitrile:methanol (v/v). The HPLC gradient was started at 30% of solvent B and 70% of solvent A and increased to 100% of B in 4 min, which was held for 2 min. The MS analysis was conducted in positive ion electrospray ionization with multiple reaction monitoring (MRM) using methods previously described^73^.

### Oxysterol quantification

Liver tissues were homogenized in PBS buffer (4x volume of tissue wet weights) using an Omni Bead Ruptor homogenizer (Omni International, Inc). Oxysterols (cholestane-3β, 5α, 6β-triol, and 7-keto-cholesterol) were extracted with 200 µL of methanol from 50 µL of the homogenate. Deuterated oxysterol compounds (7-keto-cholesterol-d7 and triol-d7, 10 ng each) were used as internal standards and were added to the samples before extraction. The calibration standards of these two oxysterols containing their deuterated internal standards were also prepared for the absolute quantification of the oxysterols in liver tissues. Oxysterols and their standards were derivatized with *N*, *N*-dimethylglycine (DMG) to increase the MS sensitivity. Sample analysis was performed with a Shimadzu 20AD HPLC system, a Shimadzu autosampler coupled to a triple quadrupole mass spectrometer (Sciex 6500+Qtrap: Applied Biosystems) operated in MRM mode. The positive ion ESI mode was used for detection of derivatized oxysterols. An Agilent C18 HPLC column (Eclipse XDB-C18 3.0 × 100 mm, 3.5 µm) was used with mobile phases (A: 1 % formic acid in water, B: 1% formic acid in 1:1 acetonitrile/methanol). The study samples were injected in duplicate for data averaging. Data processing was conducted with Analyst 1.6.3 (Applied Biosystems).

### Triglyceride quantification

1 × 10^6^ CHO and 2E4 cells were seeded in 6-cm dishes and incubated overnight in growth medium. Cells were treated for 16 h with 500 µM PALM or control BSA medium, as described above. After rinsing with PBS, cells were harvested and lysed in 5% NP-40. Lysates were incubated at 85 °C for 3 min to solubilize triglycerides. Samples were treated with lipase and total glycerol quantified using a Triglyceride Assay Kit (Abcam ab65336).

### Fatty acid uptake

Fluorescent fatty acid uptake was performed as previously described^69^. 1 × 10^6^ CHO and 2E4 cells were seeded in 6-cm dishes. The following day, cells were trypsinized and counted by trypan blue staining. 2 × 10^6^ cells were incubated at 37 °C for 1 min in fatty acid uptake solution (20 μM fatty acid-free BSA and 6 μM BODIPY-3821 (ThermoFisher D3821). Cells were washed with ice-cold PBS, pelleted, resuspended in growth medium. Mean BODIPY fluorescence was determined by flow cytometry (10^4^ cells/sample).

### ^14^C-palmitate oxidation

Palmitate oxidation rates were determined by ^14^CO_2_ capture as described previously^74^. 5 × 10^5^ CHO and 2E4 cells were seeded in T25 flasks. The next day, cells were incubated in growth medium spiked with 0.2 μCi/ml [1-^14^C]palmitate (Perkin-Elmer EC075H250UC), and flasks were sealed with rubber septa pierced by a center well device carrying Whatman chromatography paper. After 6 h, cells were quenched in 70% perchloric acid and ^14^CO_2_ was captured by adding 2 M NaOH to the chromatography paper. After 30 min, the chromatography paper was removed from flasks, placed in scintillation fluid, and incubated overnight at room temperature. ^14^CO_2_ was quantified by scintillation counting and normalized to cellular protein.

### Mitochondrial DNA copy number

Analysis of mitochondrial: nuclear DNA ratio was performed as previously described^75^.

### mTOR signaling

To assess mTOR activity in WT and 2E4 cells, 2.5 × 10^5^ cells were seeded in 6-cm dishes. The following day, cells were rinsed in PBS and incubated either in complete or serum-free culture medium overnight (16 h). Serum-starved cells were then treated with or without 250 nM insulin for 30 min prior to cell lysis and immunoblot analyses. To measure mTOR activity in WT and 2E4 cells during oxidative stress, 2.5 × 10^5^ cells were seeded in 6-cm dishes. The following day, cells were treated with 1 mM H_2_O_2_ for 16 h. To assess mTOR activity in human skin fibroblasts in the setting of SNORA73 knockdown, 1.5 × 10^5^ cells were seeded in 6 cm plates. The following day, cells were transfected with 25 nM LNA targeting GFP (control) or SNORA73 for 24 h. Cells were then rinsed twice with PBS and incubated 16 h in serum-free culture medium. The next day, serum-starved cells were treated with 100 nM insulin for 30 min prior to cell lysis. For inhibition of mTOR signaling, cells were treated with Torin 1 (250 nM) as described in figure legends. To measure mTOR activity in tissues following euthanasia, livers were rapidly excised and homogenized.

### Immunoblotting analyses

Cell lysates and tissue homogenates were prepared using RIPA buffer (50 mM Tris-Cl, 150 mM NaCl, 0.5% Nonidet P-40, 0.1% SDS) supplemented with 1X Protease Complete (Roche 11697498001) and Phosphatase Inhibitor Mini Tablets (ThermoFisher A32957). Proteins (20 μg) were separated by 4–12% Bis-Tris polyacrylamide gel electrophoresis and transferred to PVDF membranes using an iBLOT 2 system (ThermoFisher). Blots were blocked in 5% non-fat milk for 1 h at room temperature and probed overnight with antibodies against phospho-Akt (Cell Signaling 4060, 1:1000 dilution), Akt (Cell Signaling 4691, 1:1000 dilution), mTOR (Cell Signaling 2983, 1:1000 dilution), phospho-S6K (Cell Signaling 9234, 1:1000 dilution), S6K (Cell Signaling 2708, 1:1000 dilution), and GAPDH (Fisher AB2302MI; 1:10,000 dilution). Appropriate secondary antibodies conjugated to horseradish peroxidase (HRP) were used to visualize proteins in combination with HRP substrate (Jackson Immunoresearch 703-035-155 and 111-035-144, 1:10,000 dilution).

### Measurement of newly synthesized rRNA

rRNA synthesis was assessed by Click-it Nascent RNA Capture kit (ThermoFisher C10365). Cells were incubated with 0.4 mM 5-ethynyl uridine (5-EU) in growth medium for 30 min and total RNA was collected with TRIzol. Cells were washed twice with PBS and chased in normal growth medium for indicated time points. 5-EU-labeled rRNAs were chemically clicked to biotin and captured by streptavidin T1 beads. Isolated RNAs were purified and quantified by RT-qPCR.

### Ribosome analyses

1 × 10^6^ CHO and 2E4 cells were seeded in 10-cm dishes and incubated in growth medium for 24 h. Cells were treated with 10 μg/ml cycloheximide for 5 min, then trypsinized and counted using trypan blue staining. 15 × 10^6^ cells were lysed in polysome lysis buffer (20 mM Tris pH 7.2, 130 mM KCl, 10 mM MgCl_2_, 2.5 mM DTT, 0.5% NP-40, 0.2 mg/ml heparin) and loaded onto 7–47% sucrose gradients. Samples were centrifuged at 160,000 × *g* (36,000 rpm) for 3 h at 4 °C using a Beckman SW-41 rotor and Beckman L8-80M centrifuge. Gradients were fractionated using a Brandel Gradient Fractionator system with continuous monitoring of sample absorbance at 254 nm to generate polysome profiles. To assess liver ribosomes, 10-week-old ad libitum-fed male mice treated with GFP or SNORA73 LNAs for 1 week (3 injections) were injected with 100 μl of cycloheximide (20 mg/ml) in PBS by tail vein^76^. After 5 min, animals were sacrificed by cervical dislocation and livers were excised and snap-frozen in liquid nitrogen. To prepare polysome profiles, ~100 μg liver tissue was homogenized in polysome lysis buffer using a Dounce homogenizer (glass-glass). RNA content in lysates was determined and 60 μg RNA was loaded onto sucrose gradients and processed as above.

### RNA sequencing and bioinformatics

Input RNA for transcriptome studies was recovered from liver tissue lysed in polysome buffer using Trizol LS and RNeasy mini kit (Qiagen) including on-column DNase digestion. For translatome studies, RNA was recovered from corresponding polysome fractions by Trizol-LS extraction, followed by chloroform–isopropanol–ethanol precipitation, heparinase treatment (New England Biolabs, 15 min, 37 °C), and RNA cleanup step using RNA Clean & Concentrator (Zymo). Quality of RNA samples was assessed by Agilent Bioanalyzer. Sequencing libraries were prepared using Kapa mRNA Hyper Prep (Roche) according to the manufacturer’s protocol. Briefly, total RNA samples (100–1000 ng) were polyA-selected and reverse transcribed into double-stranded cDNA. High-throughput sequencing was performed using a NovaSeq 6000 Instrument (Illumina) at The Molecular Biology Core Facilities (MBCF) at Dana-Farber Cancer Institute (DFCI). RNA-seq raw reads were 100 bp reversely stranded paired-end reads.

Quality of RNA-seq reads was checked with fastqc, which computes various quality metrics for the raw reads. Reads were trimmed for adapters and filtered by sequencing Phred quality (>=Q15) by using fastp and aligned to mouse rRNA by using bowtie^77,78^. Unmapped reads were extracted by using samtools^79^. Reads were aligned to the mouse transcriptome (Ensembl version 102) using kallisto (version 0.46.2) and transcript counts were converted to gene counts using tximport^80,81^. Counts were normalized by weighted trimmed mean of M values if necessary^82^. Read counts were transformed to log 2 counts per million, their mean-variance relationship was projected, and their observational-level weights were computed with Voom^83^. Low expressing genes were filtered out by keeping genes that have counts per million (CPM) more than 0.5 in at least 4 samples. Differential gene expression was determined by performing linear modeling using limma (version 3.46.0)^84^. Statistical significance was examined by using *p*-value adjusted for multiple tests (by the Benjamini–Hochberg false discovery rate (FDR)), and genes below FDR < 0.05 were accepted as statistically significant. Gene Ontology and KEGG pathways enrichment analyses were performed using ToppFun and DAVID. ToppFun collects the content from multiple databases, including KEGG, WikiPathways, and REACTOME^85^. Bonferroni correction was applied to the analyses to select the most relevant terms.

### Statistics

For biochemical and cell biological analyses, results are expressed as mean + standard error (SE) for a minimum of three independent experiments, and each data point derives from an independent experiment. For mouse studies, results are expressed as a mean + SE for a minimum of 4 mice, and each data point derives from a different mouse. Multiple unpaired two-tailed *t*-tests with two-stage step-up method of Benjamini, Krieger, and Yuketieli, or one- or two-way ANOVA with Tukey’s or Sidak’s multiple comparison test was used for multiple comparisons, as appropriate. For directed comparisons, the statistical significance of differences in mean values was determined by a two-tailed unpaired or paired *t*-test. For all tests, *p* < 0.05 was considered significant. Data points were included unless identified as outliers based on Grubbs or Rout test. All statistical comparisons were performed using GraphPad Prism 9 for Mac OS (version 9.0.2).

### Reporting summary

Further information on research design is available in the Nature Research Reporting Summary linked to this article.

## Supplementary information

Supplementary Information

Reporting summary

## Data Availability

The data supporting the findings of this study are available within the paper and its Supplementary Information files. RNA-sequencing data have been deposited in the Gene Expression Omnibus database under accession code GSE179228. Source data are provided with this paper.

## Source data

Source Data
