# Author Correction: Loss of SNORA73 reprograms cellular metabolism and protects against steatohepatitis

**DOI:** 10.1038/s41467-021-27255-y

**Published:** 2021-11-24

**Authors:** Arthur C. Sletten, Jessica W. Davidson, Busra Yagabasan, Samantha Moores, Michaela Schwaiger-Haber, Hideji Fujiwara, Sarah Gale, Xuntian Jiang, Rohini Sidhu, Susan J. Gelman, Shuang Zhao, Gary J. Patti, Daniel S. Ory, Jean E. Schaffer

**Affiliations:** 1 Department of Medicine, Washington University in St. Louis, St. Louis, MO USA; 2 Joslin Diabetes Center, Harvard Medical School, Boston, MA USA; 3 Department of Chemistry, Washington University in St. Louis, St. Louis, MO USA

**Keywords:** Lipids, Small RNAs, Metabolic disorders

Correction to: *Nature Communications* 10.1038/s41467-021-25457-y, published online 01 September 2021.

In this article, the author names Susan J. Gelman and Gary J. Patti were incorrectly written as Susan Gelman and Gary Patti. The original article has been corrected.

