# Supplementary material for: Loss of SNORA73 reprograms cellular metabolism and protects against steatohepatitis
Source: Nat Commun. 2021 Sep 1;12:5214. doi: 10.1038/s41467-021-25457-y (PMC8410784; doi:10.1038/s41467-021-25457-y)
Supplement: Supplementary file 1 — Supplementary Information [file 41467_2021_25457_MOESM1_ESM.docx]

**Supplementary Materials for**

Loss of SNORA73 reprograms cellular metabolism and protects against steatohepatitis

Arthur C. Sletten, Jessica W. Davidson, Busra Yagabasan, Samantha Moores, Michaela Schwaiger-Haber, Hideji Fujiwara, Sarah Gale, Xuntian Jiang, Rohini Sidhu, Susan Gelman, Shuang Zhao, Gary Patti, Daniel S. Ory, and Jean E. Schaffer

**CONTENTS**

Supplementary Figures 1-7

Supplementary Tables 1 and 2

**SUPPLEMENTARY FIGURES**

**
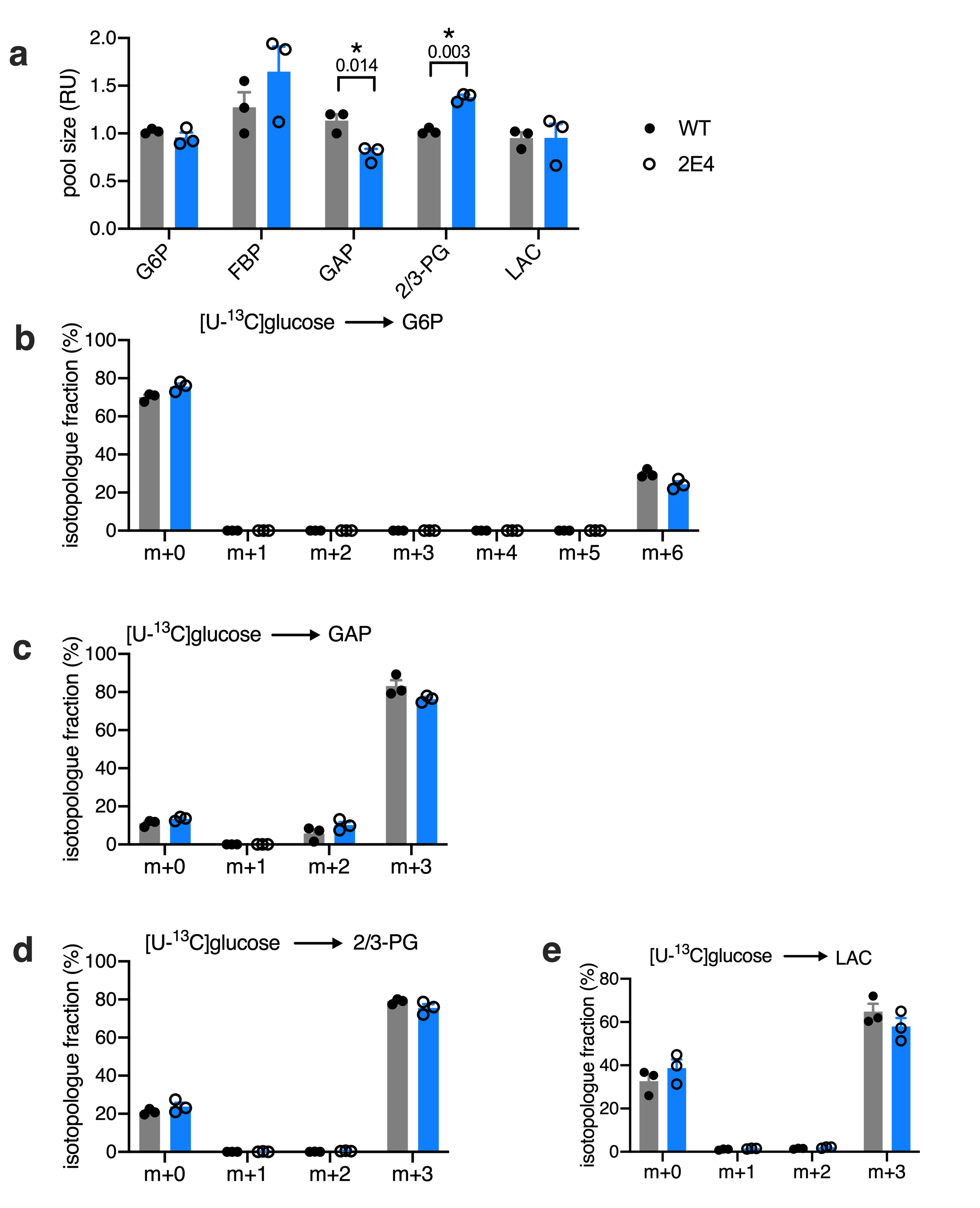
**

**Supplementary Fig. 1. Glycolysis metabolites in WT and 2E4 cells. a,** Abundance of glycolysis intermediates in WT and 2E4 cells, normalized to cellular protein content: G6P, glucose 6-phosphate; FBP, fructose 2,6-bisphosphate; GAP, glyceraldehyde 3-phosphate; 2/3-PG, 2/3-phosphoglycerate; LAC, lactate. **b**-**e**, Isotopologue distribution analysis of G6P (**b**), GAP (**c**), 2/3-PG (**d**), and LAC (**e**). Means + SE for n = 3 independent samples for each cell type. *, *p* < 0.05 for indicated comparisons (with p-values above brackets) by multiple unpaired t-test with 2-stage step-up method of Benjamini, Krieger and Yuketieli (FDR 5%). Source data are provided as a Source Data File.

**
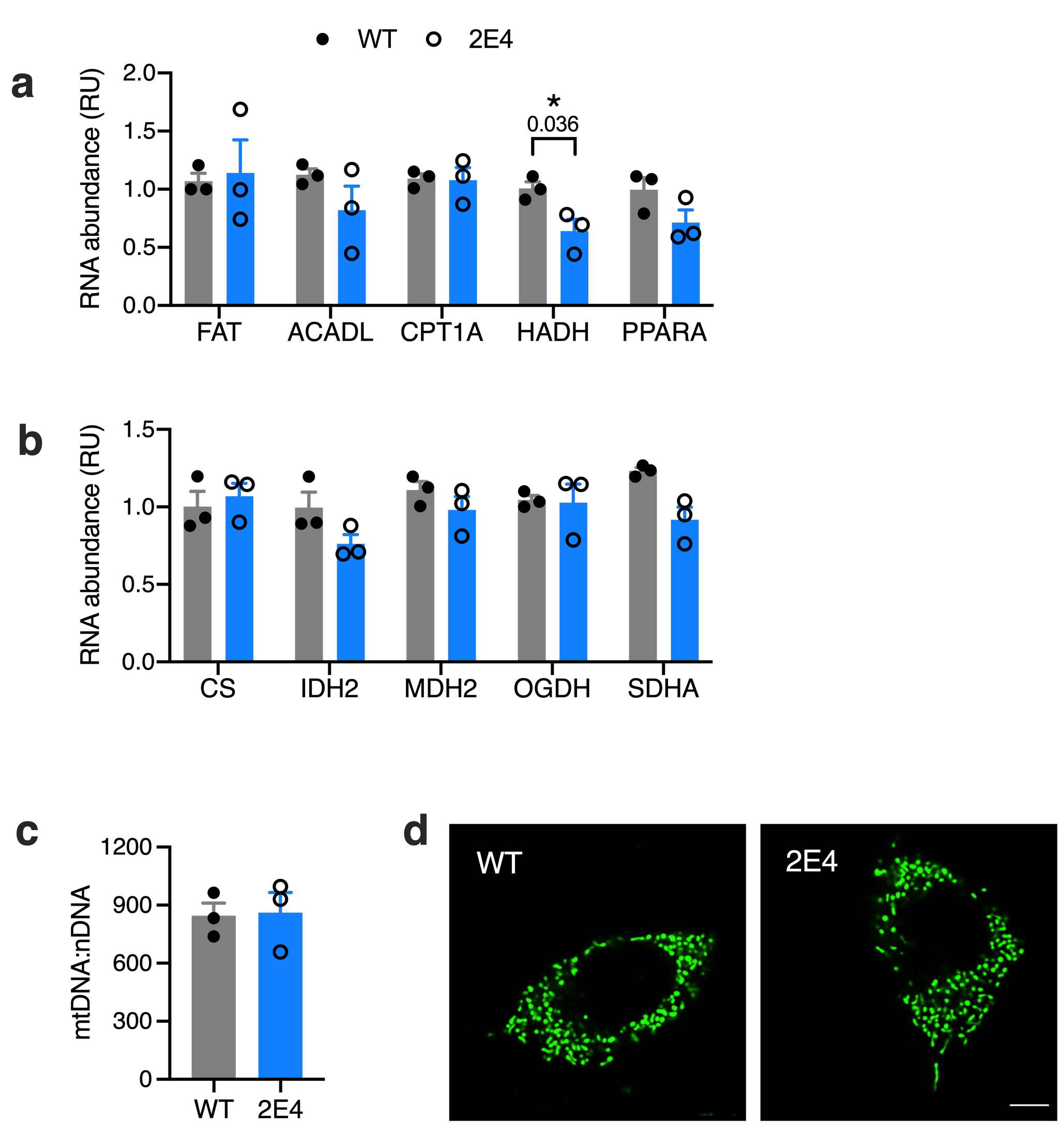
**

**Supplementary Fig. 2. Expression of TCA Cycle and β-oxidation enzymes and mitochondrial abundance in WT and 2E4 cells. a**, RT-qPCR of fatty acid oxidation genes in WT and 2E4 cells: FAT, fatty acid translocase; ACADL, acyl-coA dehydrogenase-long chain; CPT1A, carnitine palmitoyltransferase 1A; HADH, hydroxyacyl-CoA dehydrogenase; PPARA, peroxisome proliferator activated receptor-α. **b**, RT-qPCR of TCA cycle enzymes: CS, citrate synthase; IDH2, isocitrate dehydrogenase 2; MDH2, malate dehydrogenase 2; OGDH, 2-oxoglutarate dehydrogenase; SDHA, succinate dehydrogenase subunit A. **c**, Mitochondrial DNA (mtDNA) to nuclear DNA (nDNA) ratio by qPCR. **d**, Representative live cell images (3 independent experiments, 5 random cells in each of 10 random fields per cell-type-experiment) of CHO and 2E4 cells expressing GFP with mitochondrial targeting sequence. Scale bar, 5 µM. Means + SE for n = 3 independent samples for each cell type. *, *p* < 0.05 for indicated comparison (with p-value above bracket) by unpaired 2-tailed t-test. Source data are provided as a Source Data File.


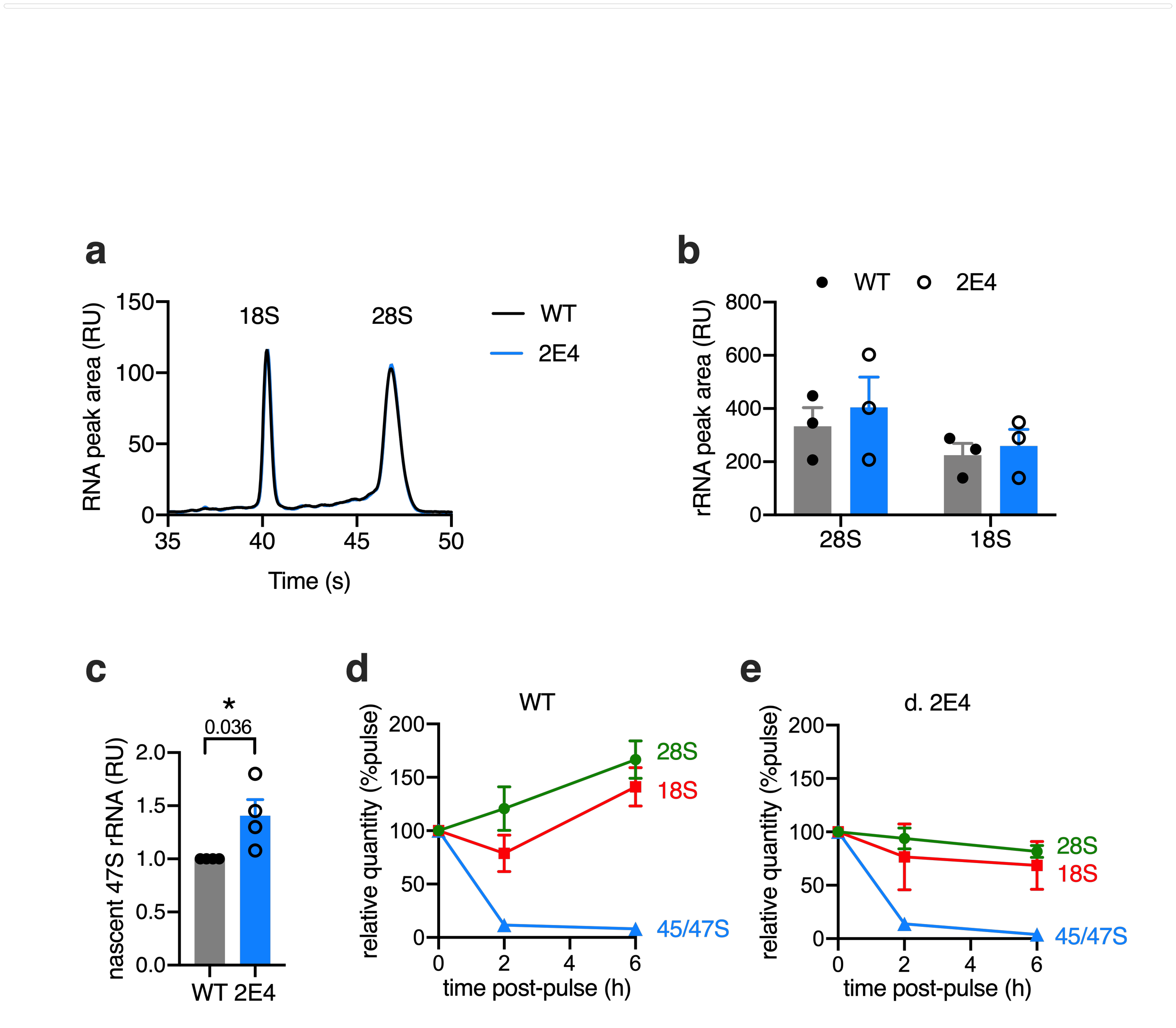


**Supplementary Fig. 3. SNORA73 haploinsufficiency alters kinetics of rRNA processing. a**-**b**, Representative bioanalyzer traces of total RNA from WT and 2E4 cells (**a**) with quantification of rRNA peak areas (**b**) from n = 3 independent experiments. **c**, RT-qPCR of 5-ethynyl uridine (5-EU)-labeled 47S rRNA species normalized to actin mRNA following a 30 min pulse with 5-EU from n = 4 independent experiments. **d-e**, RT-qPCR of 5-EU-labeled 47S, 28S, and 18S rRNA during a 6 h label-free chase in WT (**d**) and 2E4 (**e**) cells, n = 3 independent experiments. Means + SE. *, *p* < 0.05 for indicated comparisons (with p-value above bracket) by unpaired 2-tailed t-test. Source data are provided as a Source Data File.

**
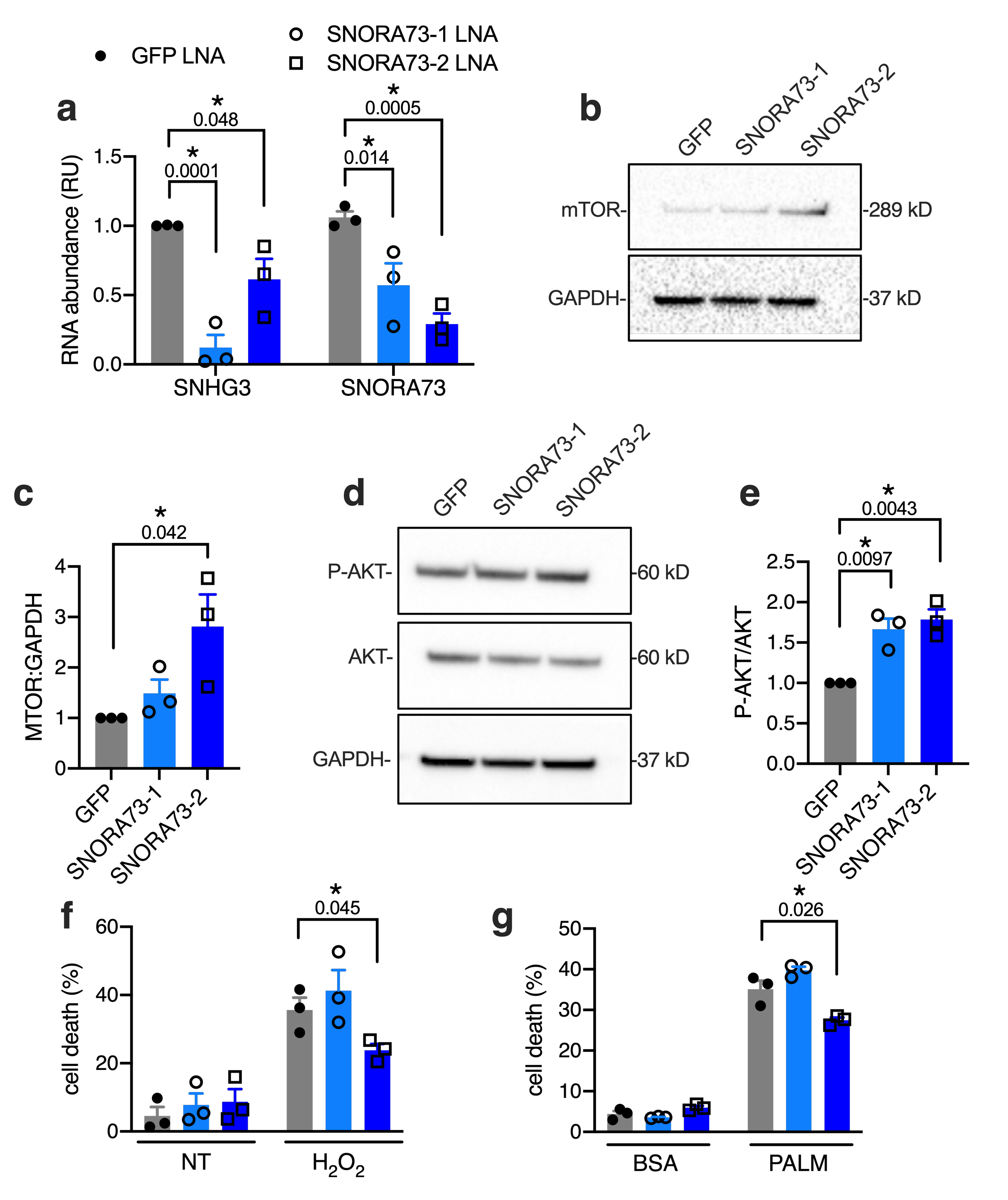
**

**Supplementary Fig. 4. SNORA73 knockdown in human cells enhances mTOR activity and protects against metabolic stress.** Normal skin fibroblasts (NSF) were transfected with control (GFP) or SNORA73 (SNORA73-1, SNORA73-2) LNAs for 24 h. **a**, RT-qPCR of SNHG3 lncRNA and SNORA73A/B. **b**-**c**, Representative immunoblot of mTOR (**b**) with quantification of n = 3 independent experiments **(c**). **d-e**, Representative immunoblot of p-AKT (S473), AKT, and GAPDH following treatment with insulin for 30 min (**d**), with quantification of independent experiments (**e**). **f-g**, Cell death following treatment with H_2_O_2_ for 16 h (**f**) or treatment with PALM or BSA for 48 h (**g**). Means + SE for n = 3 independent experiments. *, *p* < 0.05 for indicated comparisons (with p-values above brackets) by 2-way (**a**) or 1-way (**c, e**) ANOVA with Tukey’s multiple comparison test, or by unpaired 2-tailed t-test (**f, g**). Source data are provided as a Source Data file.


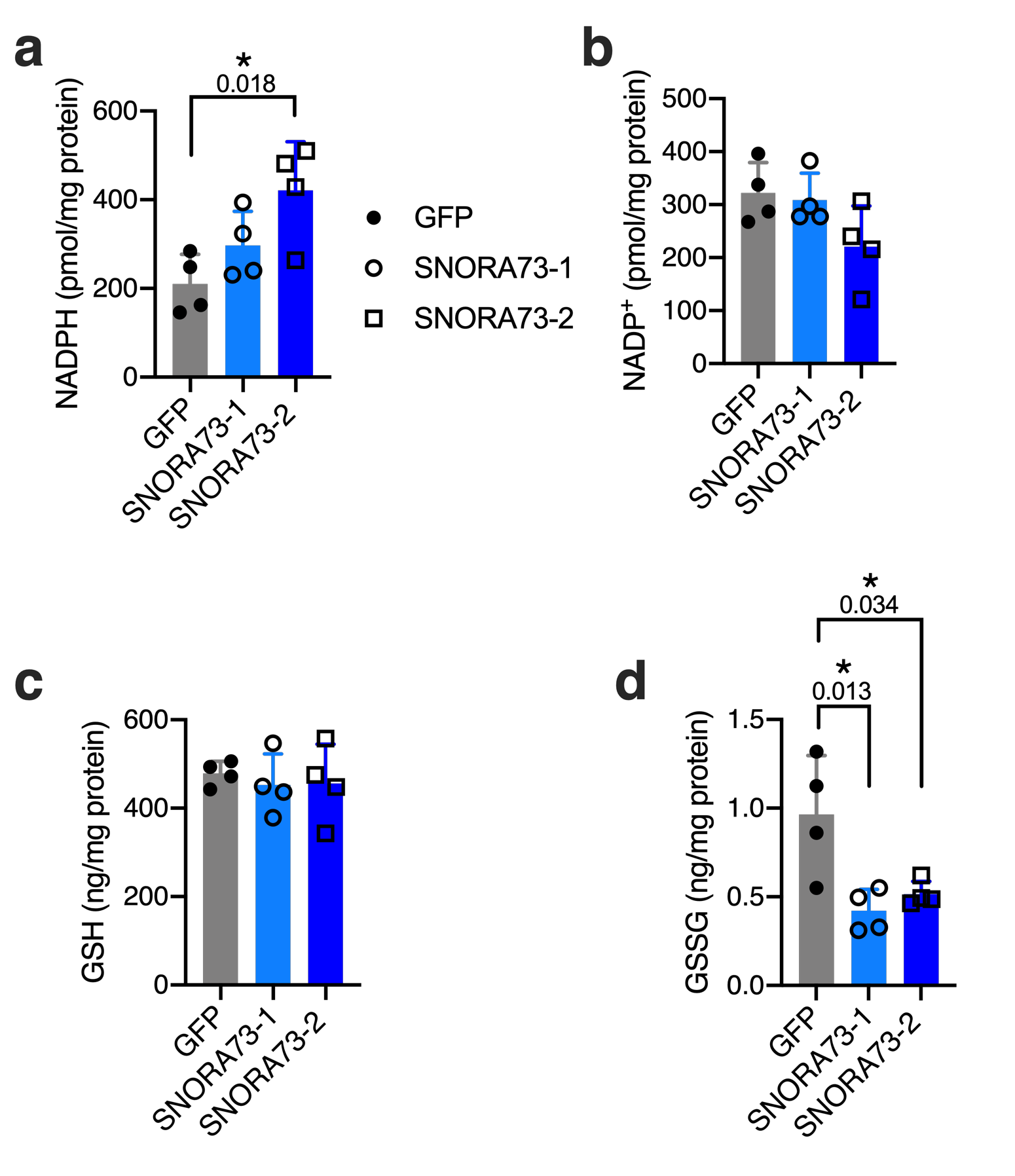


**Supplementary Fig. 5. SNORA73 knockdown in the liver alters redox state.** Analysis of livers of chow-fed mice treated with SNORA73 (SNORA73-1, SNORA73-2) or control (GFP) LNAs. **a,** NADPH. **b**, NADP^+^. **c**, GSH. **d**, GSSG. Means + SE for n = 4 mice per group. *, *p* < 0.05 for indicated comparisons (with p-values above brackets) by 1-way (**a, c, d**) or 2-way (**b**) ANOVA with Tukey’s multiple comparison test. Source data are provided as a Source Data File.

**
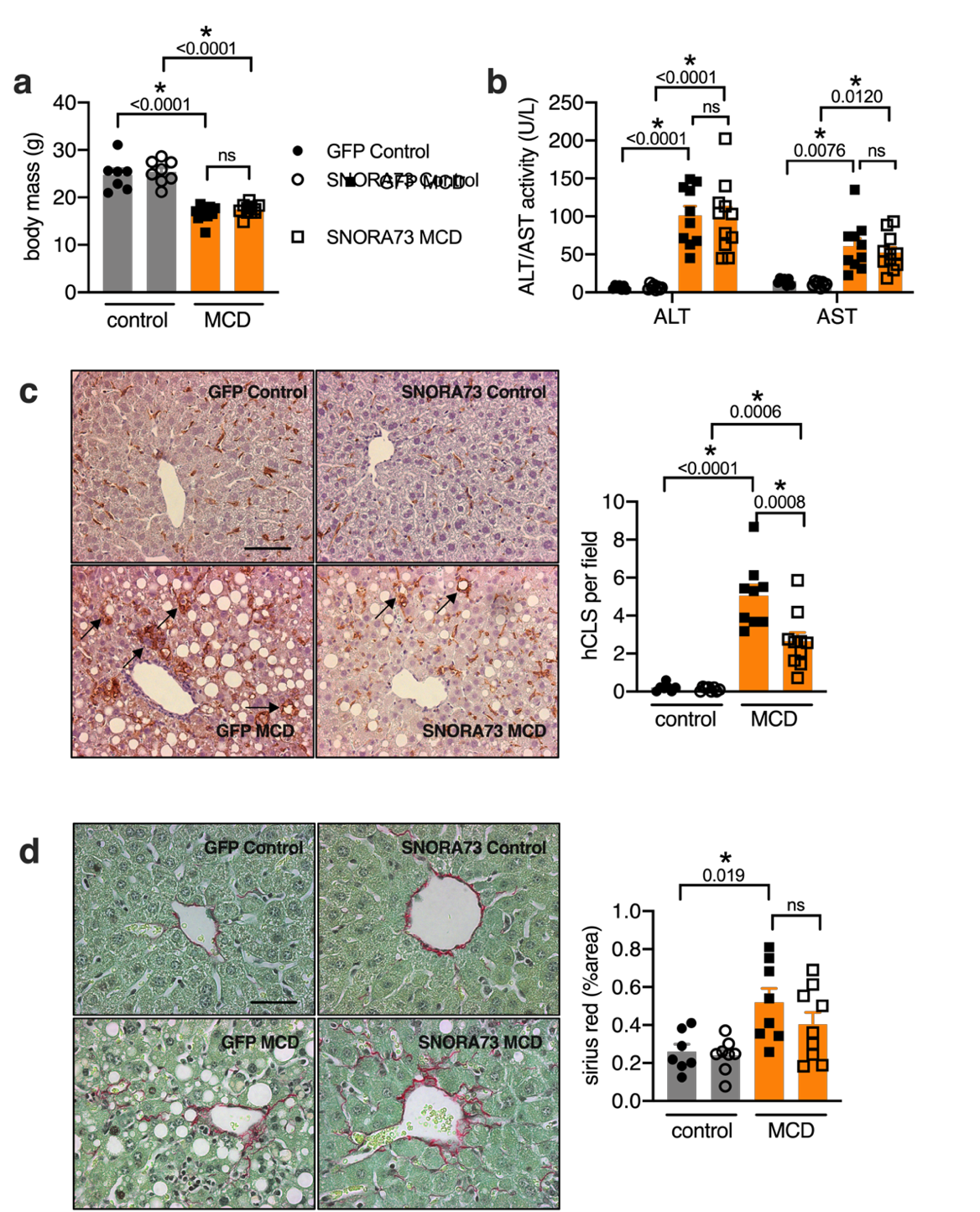
**

**Supplementary Fig. 6. Effect of SNORA73 knockdown on body weight, transaminases, inflammation, and the fibrotic response in mice challenged with MCD diet**. Mice were treated with control (GFP) or SNORA73 (SNORA73-2) LNA during feeding with control or MCD diets for 3 weeks. **a**, Body weights. **b**, Plasma alanine transaminase (ALT) and aspartate transaminase (AST). **c**-**d**, Representative images of F4/80 (**c**) and picrosirius red (**d**) staining in liver tissue with quantification of hepatic crown-like structures (hCLS, arrows) and intralobular picrosirius red staining. Scale bars = 25 μm. Means + SE. For **a- b**, GFP (n = 7) and SNORA73 (n=8) on control diet, and GFP (n=10) and SNORA73 (n = 11) on MCD diet. For **c**, GFP (n = 7) and SNORA73 (n=8) on control diet, and GFP (n=9) and SNORA73 (n=10) on MCD diet. For **d**, GFP (n = 7) and SNORA73 (n=8) on control diet, and GFP (n=8) and SNORA73 (n=9) on MCD diet. *, *p* < 0.05 for indicated comparisons by 1-way (**a, c, d**) or 2-way (**b**) ANOVA with Tukey’s multiple comparison test. ns, not significant**.** Source data are provided as a Source Data File.

**
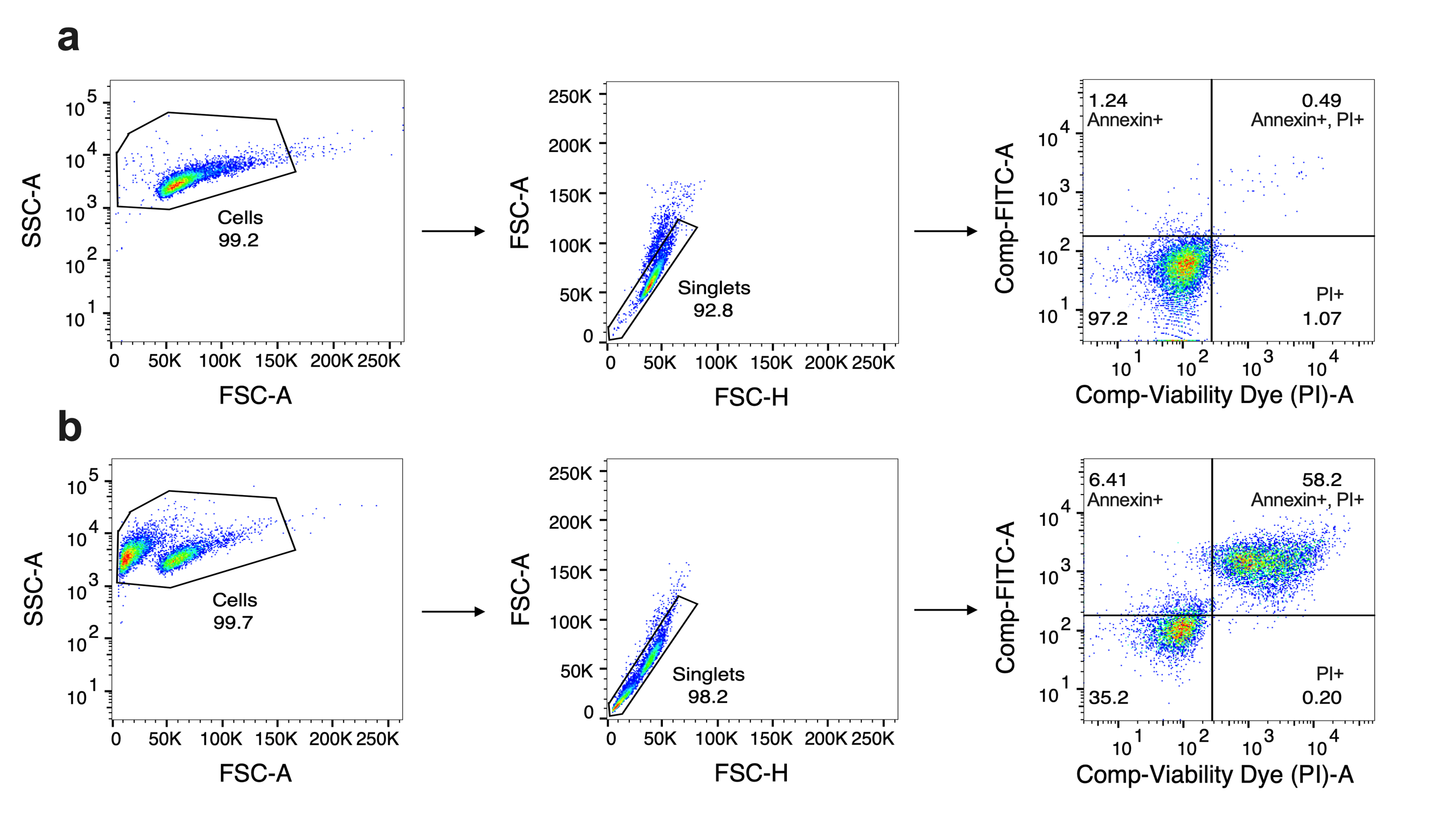
**

**Supplementary Fig. 7. Gating Strategy for Flow Cytometric Analyses.** Representative gating strategy for analyses of untreated (a) and H_2_O_2_-treated (b) WT CHO cells. Cells were initially identified by forward scatter (FSC-A) and side scatter (SSC-A), and then singlets were selected for analysis of Annexin V-EGFP (FITC-A) and propidium iodide (PI-A) staining. Quadrants were set such that <2% untreated cells were positive for either Annexin V-EGFP or PI.

**Supplementary Table 1. Changes in Transcriptome and Translatome for Selected Genes Related to Glucose or Fatty acid Metabolism or Mitochondrial Oxidative Metabolism**

| Gene Name | Transcriptome | | | Translatome | | |
| --- | --- | --- | --- | --- | --- | --- |
|  | FDR | Fold Change | P-value | FDR | Fold Change | P-value |
| Acaa2 | 0.879 | 1.04 | 0.583 | 0.755 | -1.11 | 0.438 |
| Acad9 | 0.586 | 1.19 | 0.142 | 0.584 | 1.2 | 0.222 |
| Acadl | 0.85 | -1.07 | 0.504 | 0.887 | 1.05 | 0.702 |
| Acadm | 0.5 | -1.14 | 0.0838 | 0.554 | -1.18 | 0.196 |
| Acads | 0.959 | -1.02 | 0.817 | 0.237 | -1.28 | 0.0144 |
| Acadvl | 0.898 | 1.03 | 0.628 | 0.855 | -1.05 | 0.625 |
| Aco1 | 0.676 | 1.07 | 0.224 | 0.942 | -1.02 | 0.838 |
| Aco2 | 0.257 | 1.25 | 0.0112 | 0.42 | 1.23 | 0.0877 |
| Acot1 | 0.551 | -1.54 | 0.119 | 0.407 | -1.68 | 0.0787 |
| Acot2 | 0.609 | -1.39 | 0.158 | 0.354 | -1.69 | 0.0525 |
| Aldob | 0.941 | 1.03 | 0.765 | 0.719 | 1.09 | 0.381 |
| Atp5a1 | 0.817 | 1.06 | 0.435 | 0.5 | -1.14 | 0.15 |
| Atp5b | 0.999 | -1 | 0.994 | 0.782 | -1.08 | 0.483 |
| Atp5c1 | 0.984 | 1.01 | 0.918 | 0.602 | -1.11 | 0.24 |
| Atp5d | 0.676 | 1.11 | 0.226 | 0.828 | 1.06 | 0.571 |
| Atp5g1 | 0.926 | 1.04 | 0.709 | 0.894 | -1.04 | 0.724 |
| Atp5g2 | 0.838 | -1.12 | 0.479 | 0.372 | -1.37 | 0.0606 |
| Atp5h | 0.404 | 1.13 | 0.0405 | 0.556 | 1.21 | 0.198 |
| Atp5j | 0.632 | 1.08 | 0.178 | 0.747 | 1.07 | 0.425 |
| Atp5j2 | 0.347 | 1.17 | 0.0261 | 0.789 | 1.06 | 0.496 |
| Atp5k | 0.542 | 1.12 | 0.112 | 0.503 | 1.15 | 0.152 |
| Atp5l | 0.349 | 1.17 | 0.0264 | 0.326 | 1.19 | 0.0404 |
| Atp5md | 0.817 | -1.04 | 0.434 | 0.89 | -1.04 | 0.713 |
| Atp5pb | 0.479 | -1.11 | 0.0736 | 0.274 | -1.22 | 0.0233 |
| Cd36 | 0.146 | -1.54 | 0.00295 | 0.226 | -1.75 | 0.012 |
| Cox10 | 0.457 | 1.17 | 0.0642 | 0.373 | 1.23 | 0.0608 |
| Cox11 | 0.732 | 1.08 | 0.289 | 0.838 | 1.06 | 0.589 |
| Cox15 | 0.564 | -1.16 | 0.127 | 0.379 | -1.3 | 0.0644 |
| Cox17 | 0.85 | 1.05 | 0.5 | 0.647 | 1.11 | 0.29 |
| Cox4i1 | 0.46 | 1.12 | 0.066 | 0.491 | 1.21 | 0.143 |
| Cox5a | 0.877 | 1.05 | 0.576 | 0.838 | 1.06 | 0.59 |
| Cox5b | 0.816 | 1.05 | 0.433 | 0.621 | 1.14 | 0.259 |
| Cox6a1 | 0.777 | 1.07 | 0.355 | 0.76 | -1.07 | 0.445 |
| Cox6b1 | 0.675 | 1.07 | 0.223 | 0.77 | -1.06 | 0.461 |
| Cox6c | 0.56 | 1.09 | 0.125 | 0.386 | 1.16 | 0.0689 |
| Cox7a1 | 0.471 | -1.29 | 0.0711 | 0.385 | -1.43 | 0.0682 |
| Cox7a2 | 0.966 | 1.01 | 0.838 | 0.774 | -1.06 | 0.472 |
| Cox7b | 0.888 | 1.03 | 0.606 | 0.826 | -1.04 | 0.566 |
| Cox7c | 0.756 | 1.07 | 0.324 | 0.871 | 1.04 | 0.666 |
| Cpt1a | 0.98 | -1.01 | 0.899 | 0.99 | -1 | 0.972 |
| Cpt2 | 0.998 | 1 | 0.983 | 0.89 | -1.04 | 0.713 |
| Cs | 0.934 | 1.02 | 0.734 | 0.679 | -1.09 | 0.33 |
| Cyc1 | 0.978 | -1.02 | 0.886 | 0.737 | -1.1 | 0.408 |
| Decr1 | 0.703 | 1.08 | 0.25 | 0.325 | 1.28 | 0.0397 |
| Dlat | 0.933 | 1.03 | 0.733 | 0.872 | 1.05 | 0.669 |
| Dld | 0.907 | -1.03 | 0.659 | 0.857 | -1.06 | 0.63 |
| Echs1 | 0.886 | -1.04 | 0.598 | 0.319 | -1.25 | 0.0354 |
| Eno1 | 0.818 | 1.08 | 0.437 | 0.723 | -1.09 | 0.386 |
| Fabp1 | 0.825 | 1.06 | 0.449 | 0.342 | 1.3 | 0.0471 |
| Fh1 | 0.871 | 1.04 | 0.555 | 0.974 | -1.01 | 0.924 |
| Foxo1 | 0.269 | 1.22 | 0.0131 | 0.307 | 1.26 | 0.0313 |
| Foxo3 | 0.0446 | 1.36 | 0.000327 | 0.114 | 1.56 | 0.00178 |
| Gapdh | 0.77 | 1.1 | 0.344 | 0.672 | 1.11 | 0.32 |
| Gck | 0.11 | 1.96 | 0.00182 | 0.253 | 1.92 | 0.0175 |
| Gpi1 | 0.987 | -1.01 | 0.933 | 0.994 | -1 | 0.984 |
| Hadha | 0.914 | -1.03 | 0.678 | 0.483 | -1.16 | 0.135 |
| Hadhb | 0.633 | -1.09 | 0.179 | 0.672 | -1.1 | 0.319 |
| Hnf1a | 0.781 | 1.1 | 0.366 | 0.663 | 1.15 | 0.309 |
| Hnf4a | 0.548 | 1.16 | 0.118 | 0.385 | 1.21 | 0.0676 |
| Idh1 | 0.945 | -1.02 | 0.775 | 0.702 | -1.09 | 0.358 |
| Idh2 | 0.854 | -1.07 | 0.513 | 0.679 | -1.13 | 0.33 |
| Idh3a | 0.92 | 1.04 | 0.693 | 0.763 | -1.16 | 0.449 |
| Idh3b | 0.999 | 1 | 0.992 | 0.584 | -1.12 | 0.221 |
| Idh3g | 0.995 | 1 | 0.965 | 0.823 | -1.06 | 0.561 |
| Mdh1 | 0.981 | -1.01 | 0.905 | 0.211 | -1.26 | 0.01 |
| Mdh2 | 0.934 | 1.03 | 0.734 | 0.653 | -1.13 | 0.297 |
| Mecr | 0.985 | -1.01 | 0.929 | 0.507 | -1.22 | 0.155 |
| mt-Atp6 | 0.856 | 1.06 | 0.52 | 0.276 | 1.53 | 0.0235 |
| mt-Atp8 | 0.777 | -1.18 | 0.358 | 0.583 | 1.68 | 0.22 |
| mt-Co1 | 0.918 | -1.06 | 0.688 | 0.77 | -1.13 | 0.463 |
| mt-Co2 | 0.825 | 1.05 | 0.449 | 0.587 | 1.21 | 0.224 |
| mt-Co3 | 0.298 | 1.12 | 0.0175 | 0.461 | 1.31 | 0.115 |
| mt-Cytb | 0.841 | 1.07 | 0.484 | 0.538 | 1.22 | 0.183 |
| mt-Nd1 | 0.87 | 1.06 | 0.553 | 0.335 | 1.49 | 0.0442 |
| mt-Nd2 | 0.989 | -1.01 | 0.941 | 0.669 | 1.38 | 0.317 |
| mt-Nd3 | 0.95 | -1.05 | 0.795 | 0.864 | 1.13 | 0.651 |
| mt-Nd4 | 0.955 | 1.02 | 0.807 | 0.617 | 1.29 | 0.256 |
| mt-Nd5 | 0.962 | 1.02 | 0.825 | 0.46 | 1.41 | 0.115 |
| mt-Nd6 | 0.0198 | 1.97 | 4.84E-05 | 0.108 | 1.86 | 0.00153 |
| Ndufa1 | 0.617 | 1.09 | 0.166 | 0.588 | 1.12 | 0.226 |
| Ndufa10 | 0.763 | 1.05 | 0.333 | 0.735 | 1.09 | 0.402 |
| Ndufa11 | 0.527 | 1.18 | 0.102 | 0.714 | 1.12 | 0.375 |
| Ndufa13 | 0.26 | 1.18 | 0.0119 | 0.309 | 1.22 | 0.0322 |
| Ndufa2 | 0.806 | 1.09 | 0.413 | 0.825 | 1.07 | 0.565 |
| Ndufa3 | 0.694 | 1.09 | 0.24 | 0.915 | 1.03 | 0.778 |
| Ndufa4l2 | 0.751 | 1.2 | 0.316 | 0.404 | 1.46 | 0.0772 |
| Ndufa5 | 0.83 | 1.05 | 0.46 | 0.524 | 1.17 | 0.171 |
| Ndufa6 | 0.527 | 1.11 | 0.103 | 0.521 | 1.13 | 0.168 |
| Ndufa7 | 0.641 | 1.11 | 0.186 | 0.886 | -1.03 | 0.701 |
| Ndufa9 | 0.832 | 1.07 | 0.464 | 0.589 | -1.13 | 0.228 |
| Ndufab1 | 0.967 | 1.01 | 0.843 | 0.905 | -1.03 | 0.754 |
| Ndufb10 | 0.151 | 1.23 | 0.00324 | 0.0904 | 1.48 | 0.000612 |
| Ndufb3 | 0.71 | 1.07 | 0.261 | 0.828 | 1.05 | 0.573 |
| Ndufb4 | 0.74 | 1.1 | 0.299 | 0.546 | 1.14 | 0.191 |
| Ndufb5 | 0.676 | 1.08 | 0.226 | 0.654 | 1.11 | 0.298 |
| Ndufb6 | 0.999 | 1 | 0.995 | 0.485 | -1.14 | 0.138 |
| Ndufb7 | 0.0825 | 1.33 | 0.000987 | 0.12 | 1.52 | 0.00205 |
| Ndufb8 | 0.277 | 1.19 | 0.0138 | 0.448 | 1.15 | 0.105 |
| Ndufb9 | 0.709 | 1.09 | 0.257 | 0.672 | 1.1 | 0.32 |
| Ndufs1 | 0.849 | -1.04 | 0.498 | 0.726 | -1.09 | 0.391 |
| Ndufs2 | 0.936 | 1.03 | 0.749 | 0.722 | -1.09 | 0.385 |
| Ndufs3 | 0.987 | -1.01 | 0.933 | 0.885 | -1.04 | 0.697 |
| Ndufs4 | 0.26 | 1.18 | 0.0117 | 0.246 | 1.34 | 0.0156 |
| Ndufs5 | 0.482 | 1.13 | 0.0748 | 0.445 | 1.17 | 0.103 |
| Ndufs6 | 0.74 | 1.07 | 0.299 | 0.168 | 1.33 | 0.00502 |
| Ndufs7 | 0.58 | 1.14 | 0.138 | 0.908 | 1.04 | 0.761 |
| Ndufs8 | 0.642 | 1.12 | 0.186 | 0.727 | 1.12 | 0.392 |
| Ndufv1 | 0.371 | 1.28 | 0.0327 | 0.703 | 1.11 | 0.361 |
| Ndufv2 | 0.586 | 1.08 | 0.143 | 0.883 | 1.03 | 0.691 |
| Ndufv3 | 0.571 | 1.1 | 0.132 | 0.748 | 1.07 | 0.427 |
| Nr1h3 | 0.349 | 1.18 | 0.027 | 0.579 | 1.12 | 0.216 |
| Nr1h4 | 0.715 | 1.08 | 0.267 | 0.921 | -1.03 | 0.793 |
| Ogdh | 0.302 | 1.18 | 0.0186 | 0.474 | 1.14 | 0.128 |
| Pdha1 | 0.677 | -1.09 | 0.226 | 0.861 | -1.04 | 0.647 |
| Pdhb | 0.971 | 1.01 | 0.859 | 0.904 | -1.03 | 0.752 |
| Pdhx | 0.864 | -1.05 | 0.539 | 0.676 | -1.1 | 0.326 |
| Pfkl | 0.79 | 1.08 | 0.38 | 0.943 | 1.02 | 0.84 |
| Pgam1 | 0.774 | -1.06 | 0.349 | 0.57 | -1.11 | 0.207 |
| Pgk1 | 0.953 | -1.02 | 0.8 | 0.525 | -1.12 | 0.173 |
| Pklr | 0.887 | 1.1 | 0.603 | 0.966 | 1.02 | 0.901 |
| Ppara | 0.656 | 1.24 | 0.201 | 0.539 | 1.28 | 0.185 |
| Pparg | 0.046 | -1.67 | 0.000355 | 0.114 | -1.99 | 0.00181 |
| Ppargc1a | 0.783 | 1.09 | 0.369 | 0.273 | 1.46 | 0.023 |
| Sco2 | 0.969 | 1.02 | 0.853 | 0.556 | -1.16 | 0.198 |
| Sdha | 0.979 | -1.01 | 0.897 | 0.789 | -1.05 | 0.495 |
| Sdhb | 0.642 | 1.15 | 0.187 | 0.921 | 1.03 | 0.792 |
| Sdhc | 0.754 | 1.06 | 0.321 | 0.435 | -1.16 | 0.0967 |
| Srebf1 | 0.462 | 1.24 | 0.0672 | 0.98 | 1.01 | 0.943 |
| Srebf2 | 0.872 | -1.05 | 0.562 | 0.921 | -1.03 | 0.791 |
| Sucla2 | 0.85 | -1.04 | 0.502 | 0.717 | -1.08 | 0.379 |
| Surf1 | 0.96 | -1.02 | 0.82 | 0.68 | -1.1 | 0.331 |
| Tpi1 | 0.97 | -1.01 | 0.858 | 0.74 | -1.09 | 0.412 |
| Uqcr10 | 0.89 | 1.04 | 0.612 | 0.901 | -1.03 | 0.743 |
| Uqcrb | 0.64 | 1.1 | 0.185 | 0.209 | 1.25 | 0.00974 |
| Uqcrc1 | 0.998 | -1 | 0.986 | 0.665 | -1.14 | 0.313 |
| Uqcrc2 | 0.826 | 1.05 | 0.451 | 0.879 | -1.03 | 0.68 |
| Uqcrfs1 | 0.964 | 1.01 | 0.831 | 0.871 | -1.04 | 0.666 |
| Uqcrh | 0.527 | 1.09 | 0.102 | 0.572 | 1.11 | 0.209 |
| Uqcrq | 0.472 | 1.19 | 0.0715 | 0.667 | 1.12 | 0.314 |
| Yy1 | 0.88 | -1.05 | 0.585 | 0.9 | -1.04 | 0.738 |
| Method: Input and polysome reads were normalized by trimmed mean of M-values to obtain log2 CPM. Limma-voom was used for differential expression analyses, using 2-tailed moderated t-tests, adjusted with FDR. | | | | | | |

**Supplementary Table 2. Oligonucleotide Sequences**

| **Application** | **Target** | **Oligo Sequence** |
| --- | --- | --- |
| shRNA | shSNHG3 | CAAGCACTTGTTCATAGCGGTTCTGAGTC |
| *in vitro* and *in vivo* LNA  knockdown | SNORA73 LNA 1 | +A*+A*+G*+T*+C*G*T*A*G*G*A*A*T*A*T*+G*+C*+A*+G*+G |
|  | SNORA73 LNA 2 | +G*+A*+C*+T*+C*T*G*A*G*A*A*G*T*C*G*+T*+A*+G*+G*+A |
|  | SNHG3 LNA | +T*+T*+G*+A*+C*C*G*G*G*T*C*A*A*G*A*+A*+C*+A*+A*+G |
|  | GFP LNA | +T*+C*+A*+C*+C*T*T*C*A*C*C*C*T*C*T*+C*+C*+A*+C*+T |
| RT-qPCR | Cg SNORA73 forward | GCGCCTGTATATTCCTACAGC |
|  | Mm SNORA73 forward | AACGGGAGCTTAGGGCATT |
|  | 36B4 forward | ATCCCTGACGCACCGCCGTGA |
|  | 36B4 reverse | TGCATCTGCTTGGAGCCCACGTT |
|  | SNORA73 SLRT1 | GCGTGGTCCCGACCACCACAGCCGCCACGACCACGCTTTTGT |
|  | SLRT1 reverse | TCCCGACCACCACAGCC |
|  | Mm SNHG3 forward | TCTCTCTAGGCGTCGCTCTC |
|  | Mm SNHG3 reverse | CTAATGGCCGAGGCTGTAAC |
|  | Cg SNHG3 forward | AAGAAGTCGCATCGGTACTGC |
|  | Cg SNHG3 reverse | GGTGCAAGTCCCCAAAGTTA |
|  | Cg 18S forward | AAGTGGCGTTCAGCCACCCG |
|  | Cg 18S reverse | CCAGTCAGTGTAGCGCGCGT |
|  | Cg/Mm 28S forward | TCTCGCTGGCCCTTGAAAAT |
|  | Cg/Mm 28S reverse | ACCTACATTGTTCCAACATGCC |
|  | Cg 45/47S forward | GGTCGGTGATATGGGGGAAC |
|  | Cg 45/47S reverse | TGCGTTCGAAGTGTCGATGA |
|  | Mm 18S forward | GGCCTGCGGCTTAATTTGAC |
|  | Mm 18S reverse | CGGAATTAACCAGACAAATCGC |
|  | Mm 47S forward | CACGCTGTCCTTTCCCTATTA |
|  | Mm 47S reverse | CGACAGACCCAAGCCAGTAAA |
|  | Cg CS forward | AGGTGCTGGAGTTATGGCTTG |
|  | Cg CS reverse | GGCCTGCTCCTTGGGTATC |
|  | Cg IDH2 forward | GACCTCGCGTGTTCGGGA |
|  | Cg IDH2 reverse | CGAGTCATCTCATCGCCGT |
|  | Cg MDH2 forward | GAGGTCGTTGGAGTCACTTGT |
|  | Cg MDH2 reverse | TATCGTAGAGGGTCAGGCGG |
|  | Cg SDHA forward | CGAACAGGCCACTCACTCTT |
|  | Cg SDHA reverse | GAAGTAGGTCCGCCCATAGC |
|  | Cg OGDH forward | TGCCTACCAGAGTCCCCTTT |
|  | Cg OGDH reverse | GCTACATGGTGCCCTCGTAT |
|  | Cg CD36 forward | TGCTGGCAACAAACCACAAA |
|  | Cg CD36 reverse | CTCAGTGGAAGAGCCCTAGC |
|  | Cg CPT1a forward | GAGGGCAGCCATACTCATCC |
|  | Cg CPT1a reverse | ACATCTGGACTCCCCCTCTT |
|  | Cg PPARA forward | ACTTACTGGCTTCTTCCCTTCC |
|  | Cg PPARA reverse | TGTGTCATTATTTGGGAGCTGG |
|  | Cg HADH forward | ATGGATGCTCTGTCCCTCCG |
|  | Cg HADH reverse | GAGACCAACACAGCAGACCC |
|  | Cg ACADL forward | TGCATGTTTCTTTAAGGTGTTCTC |
|  | Cg ACADL reverse | TTGCCAGCTTTCTCCCACAA |
|  | Mm COL1A1 forward | AGCACGTCTGGTTTGGAGAG |
|  | Mm COL1A1 reverse | GACATTAGGCGCAGGAAGGT |
|  | Mm COL2A1 forward | GTAACTTCGTGCCTAGCAACA |
|  | Mm COL2A1 reverse | CCTTTGTCAGAATACTGAGCAGC |
|  | Mm COL3A1 forward | CTGTAACATGGAAACTGGGGAAA |
|  | Mm COL3A1 reverse | CCATAGCTGAACTGAAAACCACC |
|  | Mm TIMP1 forward | GCAACTCGGACCTGGTCATAA |
|  | Mm TIMP1 reverse | CGGCCCGTGATGAGAAACT |
