# Supplementary material for: Loss of SNORA73 reprograms cellular metabolism and protects against steatohepatitis
Source: Nat Commun. 2021 Sep 1;12:5214. doi: 10.1038/s41467-021-25457-y (PMC8410784; doi:10.1038/s41467-021-25457-y)
Supplement: Supplementary file 2 — Reporting summary [file 41467_2021_25457_MOESM2_ESM.pdf]

nature research

Corresponding author(s): Jean E. Schaffer

Last updated by author(s): Jul 1, 2021

Reporting Summary

Nature Research wishes to improve the reproducibility of the work that we publish. This form provides structure for consistency and transparency in reporting. For further information on Nature Research policies, see our Editorial Policies and the Editorial Policy Checklist.

Statistics

For all statistical analyses, confirm that the following items are present in the figure legend, table legend, main text, or Methods section.

n/a	Confirmed	
☐	☒	The exact sample size (n) for each experimental group/condition, given as a discrete number and unit of measurement
☐	☒	A statement on whether measurements were taken from distinct samples or whether the same sample was measured repeatedly
☐	☒	The statistical test(s) used AND whether they are one- or two-sided<br>*Only common tests should be described solely by name; describe more complex techniques in the Methods section.*
☐	☒	A description of all covariates tested
☐	☒	A description of any assumptions or corrections, such as tests of normality and adjustment for multiple comparisons
☐	☒	A full description of the statistical parameters including central tendency (e.g. means) or other basic estimates (e.g. regression coefficient) AND variation (e.g. standard deviation) or associated estimates of uncertainty (e.g. confidence intervals)
☐	☒	For null hypothesis testing, the test statistic (e.g. F, t, r) with confidence intervals, effect sizes, degrees of freedom and P value noted<br>*Give P values as exact values whenever suitable.*
☒	☐	For Bayesian analysis, information on the choice of priors and Markov chain Monte Carlo settings
☒	☐	For hierarchical and complex designs, identification of the appropriate level for tests and full reporting of outcomes
☒	☐	Estimates of effect sizes (e.g. Cohen's d, Pearson's r), indicating how they were calculated

Our web collection on statistics for biologists contains articles on many of the points above.

Software and code

Policy information about availability of computer code

Data collection	No software was used.

Data analysis	5' RACE products were analyzed by NCBI BLASTN 2.2.16 [Mar-25-2007].<br>Flow cytometry analysis was performed using BD FACSDiva software [version 8.0.1 (build 2014 07 03 11 47)].<br><br>For biochemical and cell biological analyses, Methods state, "results are expressed as mean ± SEM for a minimum of 3 independent experiments. For directed comparisons, the statistical significance of differences in mean values was determined by a two-tailed or paired Student's t-test using GraphPad Prism 8 for Mac OS X (version 8.0a). p values were adjusted for multiple comparisons using the Holm-Sidak method where indicated. For all tests, $p < 0.05$ was considered significant."<br><br>For RNA sequence analyses, Quality of RNA-seq reads were checked with fastqc, which computes various quality metrics for the raw reads. Reads were trimmed for adapters and filtered by sequencing Phred quality (>= Q15) by using fastp and aligned to mouse rRNA by using bowtie (reference 77,78) . Unmapped reads were extracted by using samtools (reference 79). Reads were aligned to the mouse transcriptome (Ensembl version 102) using kallisto (version 0.46.2) and transcript counts were converted to gene counts using tximport (reference 80,81). Counts were normalized by weighted trimmed mean of M values if necessary (reference 82). Read counts were transformed to log 2 counts per million, their mean-variance relationship was projected, and their observational-level weights were computed with Voom (reference 83). Low expressing genes were filtered out by keeping genes that have counts per million (CPM) more than 0.5 in at least 4 samples. Differential gene expression was determined by performing linear modeling using limma (version 3.46.0, reference 83). Statistical significance was examined by using p-value adjusted for multiple tests (by the Benjamini-Hochberg False Discovery Rate (FDR)), and genes below FDR < 0.05 were accepted as statistically significant. Gene Ontology and KEGG pathways enrichment analyses were performed using ToppFun and DAVID. ToppFun collects the content from multiple databases, including KEGG, WikipPathways and REACTOME (reference 84). Bonferroni FDR correction was applied to the analyses to select the most relevant terms.

For manuscripts utilizing custom algorithms or software that are central to the research but not yet described in published literature, software must be made available to editors and reviewers. We strongly encourage code deposition in a community repository (e.g. GitHub). See the Nature Research guidelines for submitting code & software for further information.

Data

Policy information about availability of data

All manuscripts must include a data availability statement. This statement should provide the following information, where applicable:

- Accession codes, unique identifiers, or web links for publicly available datasets
- A list of figures that have associated raw data
- A description of any restrictions on data availability

The data supporting the findings of this study are available within the paper and its supplementary information files. RNA-sequencing data has been deposited in the Gene Expression Omnibus, accession GSE179228.

Field-specific reporting

Please select the one below that is the best fit for your research. If you are not sure, read the appropriate sections before making your selection.

☒ Life sciences ☐ Behavioural & social sciences ☐ Ecological, evolutionary & environmental sciences

For a reference copy of the document with all sections, see nature.com/documents/nr-reporting-summary-flat.pdf

Life sciences study design

All studies must disclose on these points even when the disclosure is negative.

Sample size	Sample size calculations were not performed. For cell-based studies sample size was 3-5, based upon pilot experiments that determined effect size. For some animal studies larger sample sizes were necessary because of physiological variability, based on preliminary observations of responses of C57BL6/J mice to the various diets used.
Data exclusions	Testing for outlier values was performed by Grubbs or Rout test as appropriate and outlier values were excluded as described in Methods and figure legends. Following initial analysis of 5 GFP LNA and 5 SNORA73 LNA treated livers by principal component analyses, 1 sample from each group was excluded because it failed to cluster with others in the group, leaving n = 4 for each group.
Replication	For cell-based studies, at least 3 independent experiments were performed . All findings were replicated. Attempts at replication in which control samples failed to demonstrate established findings were not further analyzed. For mouse studies all measurements are from different animals and all findings were replicated.
Randomization	Animals were randomly assigned to diets by coin flip.
Blinding	Where possible analyses were blinded (e.g., histology, analysis of tissue specimens, mass spectrometry). However, it was not possible to blind some analyses of cellular phenotypes due to limited number of persons working on studies at any one time.

Reporting for specific materials, systems and methods

We require information from authors about some types of materials, experimental systems and methods used in many studies. Here, indicate whether each material, system or method listed is relevant to your study. If you are not sure if a list item applies to your research, read the appropriate section before selecting a response.

Materials & experimental systems

n/a	Involved in the study
☐	☒ Antibodies
☐	☒ Eukaryotic cell lines
☒	☐ Palaeontology and archaeology
☐	☒ Animals and other organisms
☒	☐ Human research participants
☒	☐ Clinical data
☒	☐ Dual use research of concern

Methods

n/a	Involved in the study
☒	☐ ChIP-seq
☐	☒ Flow cytometry
☒	☐ MRI-based neuroimaging

Antibodies

Antibodies used

As provided in the methods, we used the following antibodies: phospho-Akt (Cell Signaling 4060, 1:1000 dilution), Akt (Cell Signaling 4691, 1:1000 dilution), mTOR (Cell Signaling 2983, 1:1000 dilution), phospho-S6K (Cell Signaling 9234, 1:1000 dilution), S6K (Cell Signaling 2708, 1:1000 dilution), GAPDH (Fisher AB2302MI, 1:10,000 dilution), Horseradish peroxidase-conjugated IgG (anti-chicken, Jackson Immunoresearch 703-035-155, 1:10,000 dilution), and Horseradish peroxidase-conjugated IgG (anti-rabbit. Jackson Immunoresearch 111-035-144, 1:10,000 dilution).

Validation

Validation data is provided on the Manufacturers' websites:
phospho-Akt (Cell Signaling 4060): https://www.cellsignal.com/products/primary-antibodies/phospho-akt-ser473-d9e-xp-rabbit-mab/4060
Akt (Cell Signaling 4691): https://www.cellsignal.com//products/primary-antibodies/akt-pan-c67e7-rabbit-mab/4691
mTOR (Cell Signaling 2983): https://www.cellsignal.com//products/primary-antibodies/mtor-7c10-rabbit-mab/2983
phospho-S6K (Cell Signaling 9234): https://www.cellsignal.com//products/primary-antibodies/phospho-p70-s6-kinase-thr389-108d2-rabbit-mab/9234
S6K (Cell Signaling 2708): https://www.cellsignal.com//products/primary-antibodies/p70-s6-kinase-49d7-rabbit-mab/2708
GAPDH (Fisher AB2302MI): https://www.fishersci.com/shop/products/anti-gapdh-ab-2-polyclonal-emd-millipore/AB2302MI
Horseradish peroxidase-conjugated IgG (anti-chicken): Jackson Immunoresearch 703-035-155, https://www.jacksonimmuno.com/catalog/products/703-035-155
Horseradish peroxidase-conjugated IgG (anti-rabbit): Jackson Immunoresearch 111-035-144, https://www.jacksonimmuno.com/catalog/products/111-035-144

Eukaryotic cell lines

Policy information about cell lines

Cell line source(s)

CHO-K1 (CCL-61), NIH 3T3 (CRL-1658), HEK293T (CRL-3216), and human skin fibroblast (CRL-1474) cells were from ATCC. Cell line source and numbers are provided in the Methods. The 2E4 mutant, was generated in our laboratory by introduction of the ROSA-beta-GEO retroviral promoter trap into CHO-K1 cells.

Authentication

Authentication was performed by ATCC for CHO-K1, NIH 3T3, HEK293T, and human skin fibroblast lines. Authentication was performed in our laboratory by 5'RACE and gene-walking PCR for the 2E4 mutant cell line.

Mycoplasma contamination

Cell lines were mycoplasma negative.

Commonly misidentified lines
(See ICLAC register)

No commonly misidentified cell lines were used in this study.

Animals and other organisms

Policy information about studies involving animals; ARRIVE guidelines recommended for reporting animal research

Laboratory animals

C57BL6/J male mice were purchased from Jackson Laboratories (JAX 000664). Mice were housed with a 12h:12h light:dark cycle, 70-72 degrees F and 45% humidity. Ages of animal for the different experiments are stated in the Methods and all animals were fed ad libitum. Chow diet study was performed beginning at 8 weeks of age. High fat diet study was performed beginning at 6 weeks of age. Methionine-choline-deficient diet study was performed beginning at 7 weeks of age.

Wild animals

No wild animals were used.

Field-collected samples

No field-collected samples were used.

Ethics oversight

All experimental procedures were approved by the Washington University and Joslin Diabetes Center Animal Studies Committees and conducted in accordance with the Public Health Service Policy for the Humane Care and Use of Laboratory Animals.

Note that full information on the approval of the study protocol must also be provided in the manuscript.

Flow Cytometry

Plots

Confirm that:

- [x] The axis labels state the marker and fluorochrome used (e.g. CD4-FITC).
- [x] The axis scales are clearly visible. Include numbers along axes only for bottom left plot of group (a 'group' is an analysis of identical markers).
- [x] All plots are contour plots with outliers or pseudocolor plots.
- [x] A numerical value for number of cells or percentage (with statistics) is provided.

Methodology

Sample preparation	Following various cell treatments, cells were trypsinized, pelleted and stained for annexin V-EGFP (BioVision 1004) and propidium iodide Thermo Fisher 1304MP).
Instrument	BD LSRFortessa
Software	Data were collected using BD FACSDiva software and analyzed with FlowJo v10.6.1.
Cell population abundance	Flow cytometry analysis, but not flow sorting, was performed. For each sample, 10,000 cells were analyzed .
Gating strategy	CHO cells were identified by forward scatter (FSC) and side scatter (SSC). Singlets were selected based on FSC-A, FSC-H. Quadrants were set such that <2% of untreated (healthy) cells were GFP or propidium iodide positive. Then in all samples, Annexin+ and PI+ cells were identified and quantified.

- [x] Tick this box to confirm that a figure exemplifying the gating strategy is provided in the Supplementary Information.
